# Dual-Contrast Agent with Nanoparticle and Molecular Components in Photon-Counting Computed Tomography: Assessing Articular Cartilage Health

**DOI:** 10.1007/s10439-025-03715-0

**Published:** 2025-03-28

**Authors:** Petri Paakkari, Satu I. Inkinen, Jiri Jäntti, Juuso Tuppurainen, Maria C. Fugazzola, Anisha Joenathan, Sampo Ylisiurua, Miika T. Nieminen, Heikki Kröger, Santtu Mikkonen, René van Weeren, Brian D. Snyder, Juha Töyräs, Miitu K. M. Honkanen, Hanna Matikka, Mark W. Grinstaff, Juuso T. J. Honkanen, Janne T. A. Mäkelä

**Affiliations:** 1 Department of Technical Physics, University of Eastern Finland, 70211 Kuopio, Finland; 2 Diagnostic Imaging Center, Kuopio University Hospital, Kuopio, Finland; 3 Diagnostic Center, University of Helsinki and Helsinki University Hospital, Helsinki, Finland; 4 Department of Clinical Sciences, Faculty of Veterinary Medicine, Utrecht University, Utrecht, The Netherlands; 5 Departments of Biomedical Engineering, Chemistry and Medicine, Boston University, Boston, MA USA; 6 Oulu University Hospital, Oulu, Finland; 7 Research Unit of Medical Imaging, Physics and Technology, University of Oulu, Oulu, Finland; 8 Department of Orthopaedics and Traumatology, Kuopio University Hospital, Kuopio, Finland; 9 Musculoskeletal Research Unit, University of Eastern Finland, Kuopio, Finland; 10 Department of Environmental and Biological Sciences, University of Eastern Finland, Kuopio, Finland; 11 Boston Children’s Hospital, Boston, MA USA; 12 School of Electrical Engineering and Computer Science, The University of Queensland, Brisbane, Australia; 13 Science Service Center, Kuopio University Hospital, Kuopio, Finland; 14 Radiotherapy Department, Center of Oncology, Kuopio University Hospital, Kuopio, Finland

**Keywords:** Photon-counting detector, Contrast-enhanced computed tomography, Nanoparticle, Material decomposition, Osteoarthritis

## Abstract

**Purpose:**

Photon-counting detectors (PCDs) are cutting-edge technology that enable spectral computed tomography (CT) imaging with a single scan. Spectral imaging is particularly effective in contrast-enhanced CT (CECT) imaging, especially when multiple contrast agents are utilized, as materials are distinguishable based on their unique X-ray absorption. One application of CECT is joint imaging, where it assesses the structure and composition of articular cartilage soft tissue. This evaluates articular cartilage and reveals compositional changes associated with early-stage osteoarthritis (OA) using a photon-counting detector CT (PCD-CT) technique combined with a dual-contrast agent method.

**Methods:**

A dual-contrast agent combination was used, consisting of proteoglycan-binding cationic tantalum oxide nanoparticles, developed in our lab, and a commercial non-ionic iodinated iodixanol agent. *Ex vivo* equine stifle joint cartilage samples (*N* = 30) were immersed in the contrast agent bath for 96 hours and imaged at multiple timepoints for analysis of proteoglycan, collagen, and water contents as well as collagen orientation, histological scoring, and biomechanical parameters.

**Results:**

By analyzing contrast agent concentrations, the technique provided a simultaneous assessment of the solid constituents and function of cartilage. Contrast agent diffusion depended on contrast agent composition and was significantly different between healthy and early-stage OA groups within 12 hours.

**Conclusion:**

The present study shows the promising utility of the dual-contrast PCD-CT technique for articular cartilage assessment and early-stage OA detection.

**Supplementary Information:**

The online version contains supplementary material available at 10.1007/s10439-025-03715-0.

## Introduction

Photon-counting detector (PCD) technology is revolutionizing the field of computed tomography (CT) imaging. These detectors discriminate the energy of incoming photons and enable spectral imaging with the use of a polychromatic X-ray source [1–8]. Until now, spectral imaging using conventional energy-integrating detectors (EIDs) necessitated multiple acquisitions with varying X-ray spectra or dual-layer detector setup [9–12]. However, PCD-based spectral imaging is transforming this process by capturing a complete spectral dataset in a single acquisition. PCD offers a superior signal-to-noise ratio [8, 10, 13] with a reduced radiation dose [4, 8, 12], while it eliminates the need for aligning or co-registering multiple image sets. Thus, PCD significantly streamlines both imaging procedures and subsequent analysis. PCDs are catalyzing innovations across the field of medical imaging as clinicians and researchers discover new applications. We strongly advocate that the true potential of spectral imaging will be fully harnessed in contrast-enhanced CT (CECT) applications. It will enable the simultaneous differentiation and quantification of multiple independent contrast agents within tissues or at tissue-fluid interfaces, all accomplished with just a single scan.

Assessment of articular cartilage is a compelling case example where multiple contrast agents would be of use to characterize the composition or properties of the soft tissue and its surrounding environment. Moreover, assessing the health of cartilage tissue is essential, as the degeneration of articular cartilage leads to symptoms such as joint pain, swelling, and restricted mobility, which are indicative of osteoarthritis (OA). OA is a global disease affecting an estimated 240–300 million individuals world-wide and the leading cause of disability [14]. Today, magnetic resonance imaging, ultrasound, plain radiography, and CT (alongside physical examinations) all play a pivotal role in the diagnosis and monitoring of OA [15, 16]. However, detecting early-stage OA or post-traumatic OA remains a challenge [17]. At this early stage of the disease, water content increases due to a depletion of proteoglycans and disruption in the collagen matrix architecture, leading to impaired resilience of the tissue [18].

**Fig. 1:**
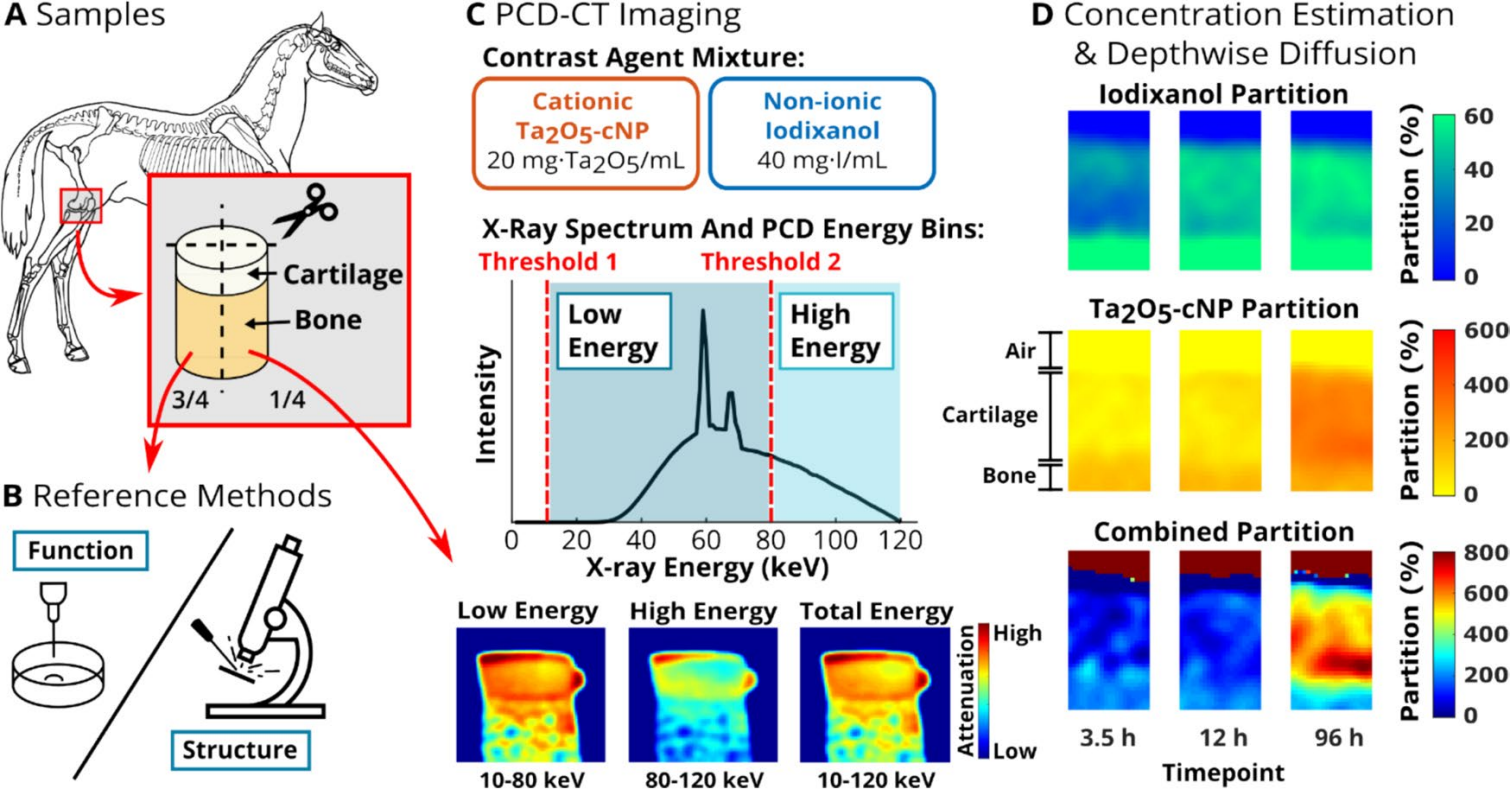
Study workflow of Part A. Equine stifle joint osteochondral plugs (*N* = 30, *d* = 8.5 mm, 15 animals) were extracted from the medial femoral condyle and the distal intertrochlear groove (one plug from both locations) (**A**). Samples were divided into quarters: three quarters for reference methods and one quarter for photon-counting detector computed tomography (PCD-CT) imaging. Articular cartilage function was determined with indentation (*i.e.*, stress-relaxation and sinusoidal loading) and structure with spectroscopical and microscopical methods (*i.e.*, Fourier-transform infrared spectroscopy, polarized light microscopy, digital densitometry, water content measurements, and Mankin scoring) (**B**). Samples were imaged with PCD-CT at multiple timepoints (0, 3.5, 6, 12, 24, 48, and 96 hours) during the diffusion of contrast agent mixture, *i.e.*, cationic tantalum oxide nanoparticle (Ta_2_O_5_-cNP) and non-ionic iodixanol (**C**). For concentration estimations, a calibration-based material decomposition was used, and it was done depthwise for each sample (**D**). Concentrations were converted to partitions (*i.e.*, measured concentration divided by the bath concentration), and combined partition was calculated (*i.e.*, Ta_2_O_5_-cNP partition divided by iodixanol partition) for final analysis

CECT enables enhanced visualization and assessment of cartilage integrity, thickness, and constituents (such as proteoglycans), and is a valuable tool for cartilage research and diagnosis [19–29]. Current contrast agents include small molecule anionic [30], non-ionic [20], and cationic [31] iodinated compounds as well as novel tantalum- [24, 32, 33], bismuth- [34], and gold-based [35, 36] nanoparticle contrast agents, each of which has been fine-tuned for imaging a specific cartilage characteristic. Given our interest in developing dual-contrast CECT technique for PCD-CT, we require two contrast agents, which are spectrally resolved and target different cartilage structural or functional properties. Herein, we report a novel dual-contrast cartilage imaging technique employing cationic tantalum oxide nanoparticles (Ta_2_O_5_-cNPs, Grinstaff Group, Boston University, Boston, USA) [24, 32] and non-ionic iodinated iodixanol (Visipaque™, GE HealthCare, Chicago, USA) (Fig. 1). This approach enables the assessment of solid constituents and porosity of articular cartilage, allowing for the determination of crucial biomechanical properties such as load-bearing capacity, elasticity, and resistance to mechanical stresses. Unlike previous studies that utilized cationic iodinated CA4+ and non-ionic gadolinium-based gadoteridol [27, 28, 37–41], our technique features a significantly larger* K*-edge difference between the iodine and tantalum (33.2 keV and 67.4 keV, respectively) contrast agents compared to the gap between the *K-*edges of iodine and gadolinium (33.2 keV and 50.2 keV, respectively). Another major difference is the relatively large size of the nanoparticles (diameter: approx. 2-3 nm) compared to conventional molecular compounds, which is significant when compared to conventional molecular compounds, as this size may interact more effectively with collagen structures, potentially revealing their properties and behavior. Specifically, we conduct CECT imaging of osteochondral cartilage samples utilizing experimental PCD-CT setup in Part A and commercial EID-based microCT in Part B, combined with a dual-contrast agent method utilizing cationic Ta_2_O_5_-cNPs and non-ionic iodixanol. We hypothesize that (1) the combination of nanoparticle and molecular contrast agents will provide assessment of articular cartilage structure and function, and enable differentiation of healthy and early-stage OA cartilage explants *ex vivo* with a single scan using PCD-CT (Part A); and (2) the interaction between nanoparticle and molecular contrast agents influences their diffusion dynamics within cartilage tissue (Part B).

## Materials and Methods

A brief presentation of the materials and methods is given here. More details are presented in the Supplementary Material.

### Samples and Sample Processing

Part A: Cylindrical osteochondral samples (*N* = 30, *d* = 8.5 mm) were collected from 15 stifle joints of 15 healthy mature Criollo horses (livestock from an abattoir, no sample-related confounders were controlled but the age range was from four to six years old), with the joint randomly selected for each animal. From each horse, two samples were harvested: one from the distal intertrochlear groove and one from the medial femoral condyle. Each sample was split into halves and one half was used for histological reference methods and the other half was halved once more to create two quadrants: one quadrant was used for determining water content and the other for contrast agent diffusion measurements.

Part B: Cylindrical osteochondral samples (*N* = 4, *d* = 8.5 mm) were collected from four stifle joints (two left and two right joints) of two healthy mature Dutch Warmblood horses( euthanized mares, aged 11 and 17 years old). From each joint, one sample was harvested: the lateral trochlear ridge samples were collected from one horse (one per side) and the medial femoral condyle samples were collected from another horse (one per side). Each sample was split into halves and one half was not used in this study and the other half was halved once more to create two quadrants which were used to form two identical sample groups for the contrast agent diffusion test.

The osteochondral plugs were harvested using a hollow drill reaching depth of approximately 10 mm; thus, the samples included articular cartilage, subchondral, and trabecular bone. A razor blade and a hammer, guided by a metallic jig, were used to cut the samples into halves or quarters to ensure even sized halves and quarters. The samples were preserved frozen (− 20 °C) in vials filled with phosphate-buffered saline (PBS). To allow the contrast agent diffusion only through the articulating surface, the bottom and sides of the diffusion samples were sealed using cyanoacrylate (Loctite, Connecticut, USA).

### Contrast Agent Solutions

The dual-contrast agent bath used in the study contained 20 mg·Ta_2_O_5_/mL and 40 mg·I/mL of Ta_2_O_5_-cNP and iodixanol, respectively. Additionally, in Part B, the single-contrast agent baths used the same concentrations but the bath contained only one contrast agent at the time. Different solutions were also made to calibrate and validate the calibration-based material decomposition method. The calibration solutions were single-contrast agent solutions and included concentrations of 10, 20, 40, 80, 120, and 160 mg·Ta_2_O_5_/mL for Ta_2_O_5_-cNP and 5, 10, 20, 30, 40, and 50 mg·I/mL for iodixanol. The validation solutions were mixtures with varying concentrations of Ta_2_O_5_-cNP and iodixanol: 20/20, 80/20, 140/20, 20/40, 80/40, 140/40, 20/80, 40/80, and 60/80 mg·(Ta_2_O_5_/I)/mL. The Ta_2_O_5_-cNP was synthesized as described earlier [32]. All the solutions were prepared each in its own vial (approx. volume of 2 mL) and the osmolality of the mixture was set to 320 m·Osm/kg to mimic the osmolality of a saline solution. The sample baths also contained PBS with protease inhibitors (ethylenediaminetetra-acetic acid disodium salt dihydrate [*C* = 1.86 g/L, VWR International, Radnor, PA, USA] and benzamidine hydrochloride hydrate [*C* = 0.78 g/L, Sigma-Aldrich Co., St. Louis, MO, USA]) to prevent degradation of the samples.

### PCD-CT Setup

In Part A, the experimental PCD-CT setup consisted of two-bin PCD (XC-Flite FX15, Xcounter AB, Stockholm, Sweden), mini-focus X-ray source (IXS1203MF, VJ X-Ray, Bohemia, USA), and motorized rotator (NR360S, Thorlabs Inc., Newton, USA). The X-ray source voltage and current were set to 120 kVp and 0.25 mA, respectively (Fig. 1C), and strong filtering was applied (3.0 mm aluminum and 0.5 mm copper). The reconstructed voxel size was 68.4 × 68.4 × 68.4 µm^3^.

The PCD used in our study experienced some electrical drift in higher energies, leading to detected photons having higher energy readings than their actual values [42]. To account for this, the higher threshold was set at 80 keV, as opposed to 70 keV, which would be closer to the tantalum K-edge (*i.e.*, 67.4 keV). The lower energy threshold of the PCD was set at 10 keV to filter out electrical noise. This energy bin selection was validated through preliminary imaging experiments, where calibration and validation solutions were scanned using both 10/70 keV and 10/80 keV binning. The 10/80 keV combination resulted in lower errors in concentration estimation. Consequently, the captured energy bins were 80–120 keV for high energy bin and 10–120 keV for the total energy bin, while the low energy bin (10–80 keV) was calculated offline.

### Dual-Energy MicroCT Setup

In Part B, the imaging was conducted with a dual-energy protocol using conventional EID microCT (Nikon XT H 225, Nikon Metrology, Leuven, Belgium). The low/high energy settings were 80/130 kVp, 312.5/230.8 µA, and 2.0 mm aluminum/1.5 mm copper filters for X-ray source voltage, current, and beam filtering, respectively. The reconstructed voxel size was 25.0 × 25.0 × 25.0 µm^3^.

### Imaging Protocol

Part A: The calibration and validation solution vials were imaged individually (*i.e.*, one solution vial at a time) with PCD-CT to minimize artifacts, such as beam hardening. The osteochondral samples were thawed overnight in PBS-filled vials at 8 °C and imaged in groups of four (except the last group which only had two samples), totaling eight groups. To follow the diffusion, the samples were imaged at seven different timepoints: 0, 3.5, 6, 12, 24, 48, and 96 hours, chosen based on prior single-contrast agent studies [43, 44]. The samples were inside an airtight plastic container to avoid drying during the imaging. Image acquisition took 12.5 minutes, and the total time the samples were out of the solution was approximately 20 minutes per timepoint. The contrast agent bath was constantly shaken to ensure even distribution of contrast agents, and its temperature was kept at 37 °C during the diffusion to simulate human body temperature.

Part B: The calibration solutions were imaged with EID-CT in two batches: one had all the iodixanol solutions and the other all the Ta_2_O_5_-cNP solutions. The validation solutions were also divided into two batches containing four and five mixtures each. Similarly to Part A, the samples were thawed overnight in PBS-filled vials at 8 °C. Part B included single and dual-contrast agent solutions, and the samples were split into two sample-wise identical study groups: Group 1 and Group 2. The Group 1 samples were first placed in a single-contrast agent bath containing only iodixanol and imaged at 0, 3, 6, 12, 24, 48, and 72-hour timepoints to follow the iodixanol’s diffusion into native cartilage. Then, the Group 1 samples were placed in a dual-contrast agent bath containing iodixanol and Ta_2_O_5_-cNP to follow Ta_2_O_5_-cNPs’ diffusion subsequent to iodixanol diffusion and imaged at 3, 6, 12, 24, 48, 72, 96, 120, 143, and 165-hour timepoints (when earlier diffusion is not counted). The Group 2 samples were first placed in a single-contrast agent bath containing only Ta_2_O_5_-cNPs and imaged at 0, 3, 6, 12, 24, 48, 72, 96, 120, and 144-hour timepoints to follow the Ta_2_O_5_-cNPs’ diffusion into native cartilage. Then, the Group 2 samples were placed in a dual-contrast agent bath to follow the iodixanol’s diffusion subsequent to Ta_2_O_5_-cNPs diffusion and imaged at 3, 6, 12, 24, 48, 71, and 93-hour timepoints. The samples were inside an airtight plastic container to avoid drying during the imaging. Imaging time was about 13 minutes, while the total time the samples were out of the solution was approx. 40 minutes per timepoint. Total diffusion time for both groups was 237 hours (not including handling time). Similarly to Part A, the contrast agent bath was constantly shaken, and its temperature was kept at 37 °C during the diffusion.

### Quantitative Assessment of Contrast Agent Concentrations in Cartilage

**Fig. 2:**
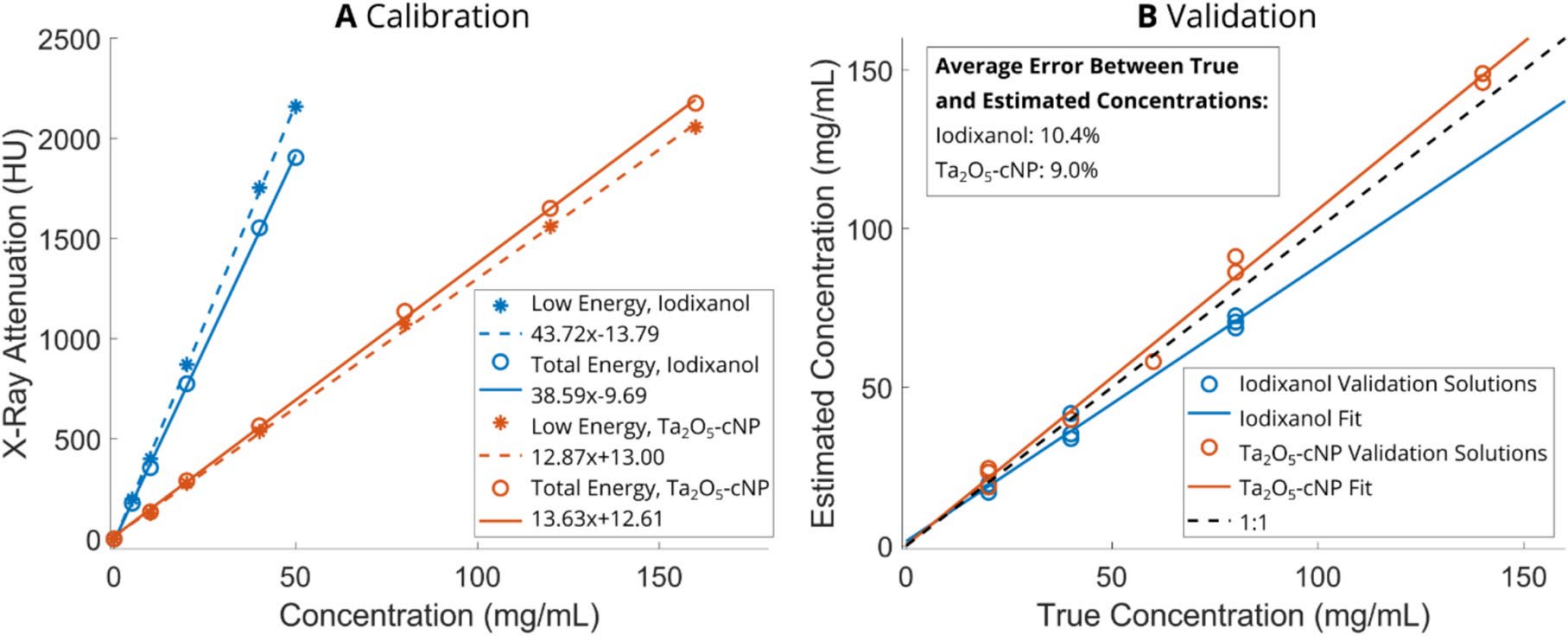
Calibration and validation curves of Part A. In subfigure **A**, the X-ray attenuation is presented as a function of contrast agent concentration at low (10–80 keV) and total (10–120 keV) energy bins. The calibration concentrations were between 10 and 160 mg·Ta_2_O_5_/mL for cationic tantalum oxide nanoparticles (Ta_2_O_5_-cNPs) and 5–50 mg·I/mL for iodixanol since the highest anticipated concentration was estimated to be in these ranges. In subfigure **B**, the determined relationship between contrast agent concentration and X-ray attenuation in Hounsfield units (HUs) was applied when validating the capability of the technique to determine composition of contrast agent mixtures. The concentrations in solutions were between 20 and 140 mg·Ta_2_O_5_/mL for Ta_2_O_5_-cNP and 20-80 mg·I/mL for iodixanol. Dashed black line represents the optimal concentration estimation and error in legend is the average error between true concentrations and estimated concentration

In both parts of this study, the contrast agent concentrations were computed based on the calibration experiments (only PCD-CT data are shown, Fig. 2A). Subsequently, the fitted calibration coefficients (slope values) were validated against validation solutions (only PCD-CT data are shown, Fig. 2B). The estimated concentrations were transformed to contrast agent partitions by dividing the estimated concentration by the concentration of the original bath, providing a measure of how much of each contrast agent has diffused into the cartilage compared to the initial amount [40]. Additionally, in Part A, a combined partition was calculated by dividing the Ta_2_O_5_-cNP partition by iodixanol partition. This combination has been shown to strengthen the correlation with cartilage composition especially in early timepoints with cationic iodinated CA4+ and non-ionic gadolinium-based gadoteridol [27, 28, 37].

The diffusion parameters of the contrast agent diffusion were calculated by fitting the following polynomial equation to the bulk partitions of the contrast agents at each timepoint [23, 45]:

1 $$P_{{{\text{Diff}}}} \left( t \right) = P_{{{\text{Max}}}} \cdot \left( {1 - e^{{\frac{t}{ - \tau }}} } \right) ,$$

where *P*_Diff_ is the measured diffusion partition, *P*_Max_ is the maximum partition, *t* is a timepoint, and *τ* is the diffusion time constant when 63.2% of the maximum partition of the contrast agent is reached [23, 45]. The *P*_Max_ helps to assess the contrast agent partition at diffusion equilibrium and the *τ* measures the contrast agent flux during the diffusion.

### Mechanical Testing and Analysis

A four-step biomechanical stress-relaxation indentation protocol was conducted for the samples of Part A similarly to our previous works [43, 46]. The equilibrium modulus (*E*_Eq_) was calculated from the stress–strain ratio at the mechanical equilibrium and the instantaneous modulus (*E*_Inst_) was calculated immediately after the ramp phase of the step. Stress-relaxation time constant *α* and stretching parameters *β* were determined by fitting the following equation to the relaxation phase of the 2^nd^ step [47]:

2 $$f_{{{\text{relax}}}} \left( t \right) = a \cdot e^{{ - \left( {t/\alpha } \right)^{\beta } }} + k ,$$

where* a* and *k* are fitting constants, and *t* is the time. The dynamic modulus (*E*_Dyn_) was defined from the ratio of stress and strain amplitudes of the sinusoidal data, while the phase shift (*θ*) between the stress and strain amplitudes was calculated using Fourier transform [46]. The effect of the indentation geometry on the results was corrected by using the solution derived by Hayes et al. [48]. Estimated Poisson’s ratios used in the calculations were 0.2 for the *E*_Eq_ and 0.5 for the *E*_Inst_ and *E*_Dyn_ [49].

### Microscopic and Spectroscopic Characterization of Cartilage Composition

Fourier-transform infrared spectroscopy, polarized light microscopy, and digital densitometry methods were used for the determination of collagen and proteoglycan contents, collagen fiber network orientation, and parallelism index of the samples in Part A, similarly as in our previous works [43, 46]. The blind-coded fixed histological sections were also graded by four independent assessors based on the Mankin scoring system [50]. Then, the samples were categorized into two groups based on Mankin scoring and using grade 1.5 as a divider: healthy (Mankin grades < 1.5, *n* = 10) and early-stage OA (Mankin grades 1.5–4, *n* = 20) categories. This classification reflects subtle degenerative changes that are not yet detectable in clinical imaging but indicate early-stage cartilage deterioration. Thus, some horses could have combination of healthy and early-stage OA cartilage in the same joint. The depthwise water content was determined in 200-µm-thick sections through the cartilage by weighing the section before and after freeze-drying.

### Statistical Analysis

As data could not be assumed normally distributed, Spearman’s rank correlation coefficient was used to evaluate the relationship between the changes observed in the contrast agent partition data and the corresponding reference data. Mann-Whitney *U* test was used to check statistical difference between Mankin groups (*i.e.*, healthy and early-stage OA groups). The statistical analyses were performed with MATLAB (R2021b, MathWorks, Natick, MA, USA) using Statistics and Machine Learning Toolbox (version 12.2).

## Results

### Part A: Sensitivity of the Dual-Contrast Agent Method

#### Simultaneous Estimation of Contrast Agent Concentrations Using PCD-CT

The PDC-CT calibration coefficients for contrast agent concentration estimation for each energy bin are presented in Figure 2A (high energy data not shown). The energy bin combination with the smallest error in validation solutions was with low and total energy bins (Fig. 2B). The iodixanol concentration was underestimated, on average, by 10.4%, signifying that the material decomposition method tends to estimate lower iodixanol concentrations than the actual ones. Conversely, the Ta_2_O_5_-cNP concentration was overestimated by an average of 9.0%.

#### Depthwise Analysis of Contrast Agent Specific Distribution

**Fig. 3:**
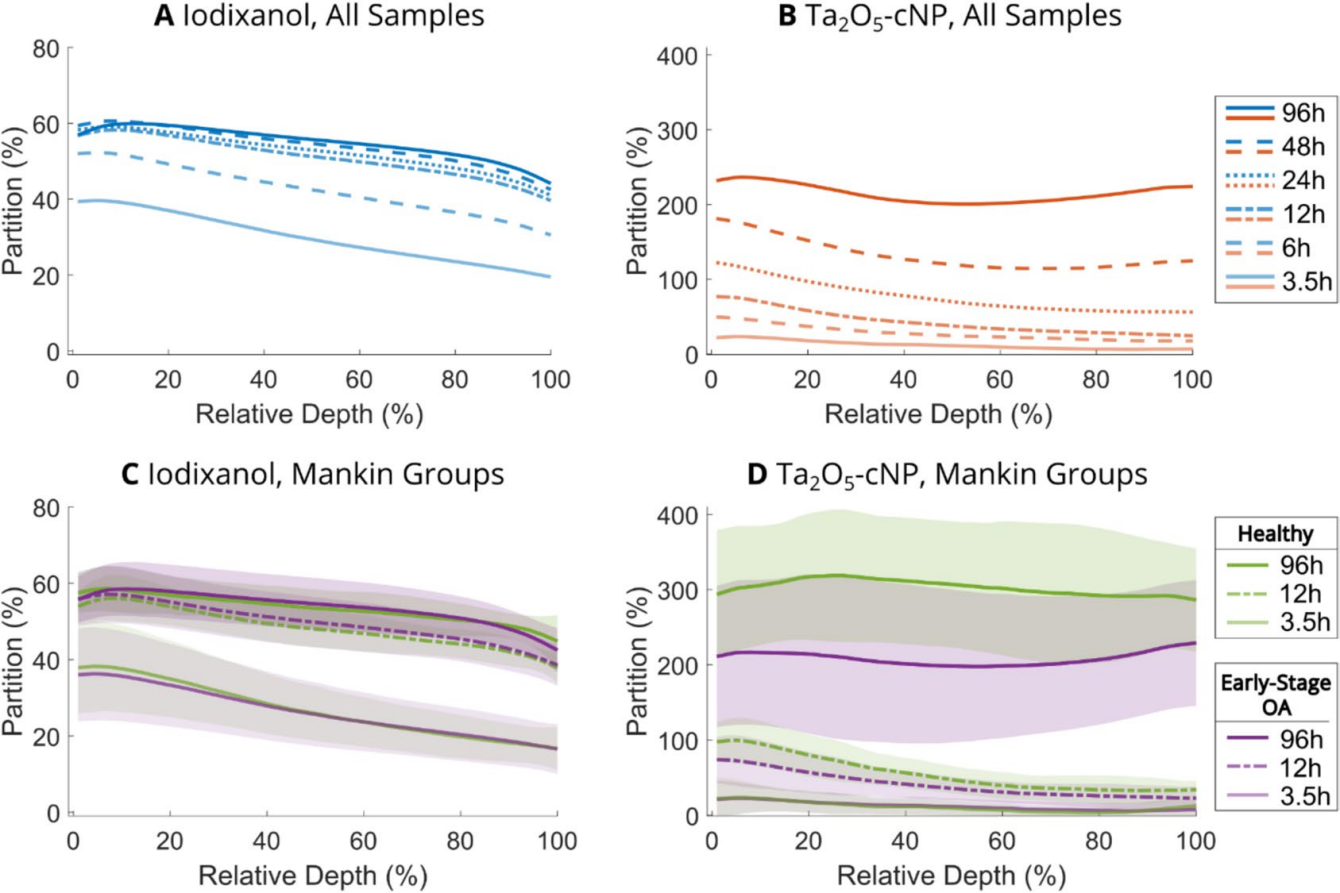
Averaged depthwise profiles of the contrast agents for all samples (subfigures **A** and **B**) and for samples categorized based on Mankin scoring (subfigures **C** and **D**): healthy cartilage (Mankin grades < 1.5, *n* = 10) and early-stage osteoarthritis (OA) cartilage (Mankin grades 1.5–4, *n* = 20). Subfigures **A** and **C** show profiles for iodixanol and subfigures **B** and **D** cationic tantalum oxide nanoparticle (Ta_2_O_5_-cNP). The averaged profiles in subfigures **A** and **B** represent data from all the timepoints without standard deviation. The subfigures **C** and **D** include averaged profiles of the contrast agents based on Mankin scoring with standard deviation (marked with the shaded area), but only specific timepoints are shown: 3.5-hour (the first measured timepoint), 12-hour (the first timepoint where healthy and early-stage OA groups can be distinguished with Ta_2_O_5_-cNP), and 96-hour (the last measured timepoint). The significant differences between the healthy and early-stage OA cartilage were detected at relative depths of 0–56% and 0–84% at the 12-hour and 96-hour timepoints, respectively. The articular cartilage thickness was linearly interpolated to ensure consistent thickness across all samples, where the cartilage surface is at 0% and the cartilage-bone interface at 100%

A depthwise inspection of the contrast agent accumulation inside cartilage (Fig. 3) revealed a descending partition profile of iodixanol from the surface toward the bone (Fig. 3A). The depthwise partition profiles of Ta_2_O_5_-cNP inside cartilage initially exhibited a descending trend toward the deep zone at the early timepoints. However, the Ta_2_O_5_-cNP partition continued to increase, especially in the deep zone, *i.e.*, after a relative depth of 50-60% (Fig. 3B), thus, at the 96-hour timepoint, the superficial and deep zones displayed similar Ta_2_O_5_-cNP partition levels. The depthwise differences between the Mankin groups (*i.e.*, healthy and early-stage OA cartilage) were also investigated for both contrast agents (Fig. 3C and D) and these results are reported in the section “*Distinction Between Healthy and Early-Stage OA Samples*.”

#### Contrast Agents Correlations with Articular Cartilage Composition

The iodixanol bulk partition displayed a robust negative correlation with proteoglycan content and a weak-to-moderate negative correlation with collagen angle, persisting from 3.5 hours up to 96 hours (Table 1). Conversely, the Ta_2_O_5_-cNP bulk partition exhibited a weak-to-moderate positive correlation with proteoglycan content, starting at the 12-hour mark, and a weak-to-strong negative correlation with Mankin scoring from the 24-hour point (Table 1). Comparatively, the quotient or combined partition demonstrated stronger correlation coefficients for both proteoglycan content and collagen angle in contrast to the Ta_2_O_5_-cNP partition alone. Notably, the correlation with proteoglycan content became significant at the 12-hour timepoint, while the correlation with collagen angle achieved significance at the same 12-hour mark (Table 1).

**Table 1:**
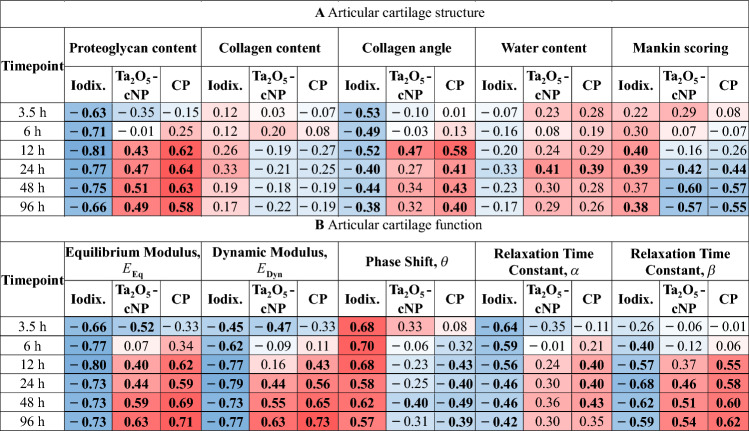
Spearman’s correlation coefficient between contrast agent bulk partitions and articular cartilage structure (**A**) and function (**B**) as a function of diffusion time

The values of contrast agent partitions and articular cartilage function were converted to bulk values before calculating correlation coefficients. Combined partition (CP) is calculated by dividing tantalum oxide nanoparticle (Ta_2_O_5_-cNP) partition by iodixanol (Iodix.) partition. Statistically significant (*p* < 0.05) values are bolded, and the sign correlation coefficient is signified with blue (negative) and red (positive) color and the strength of correlation coefficient with color tone (darker color equals stronger correlation coefficient)

**Fig. 4:**
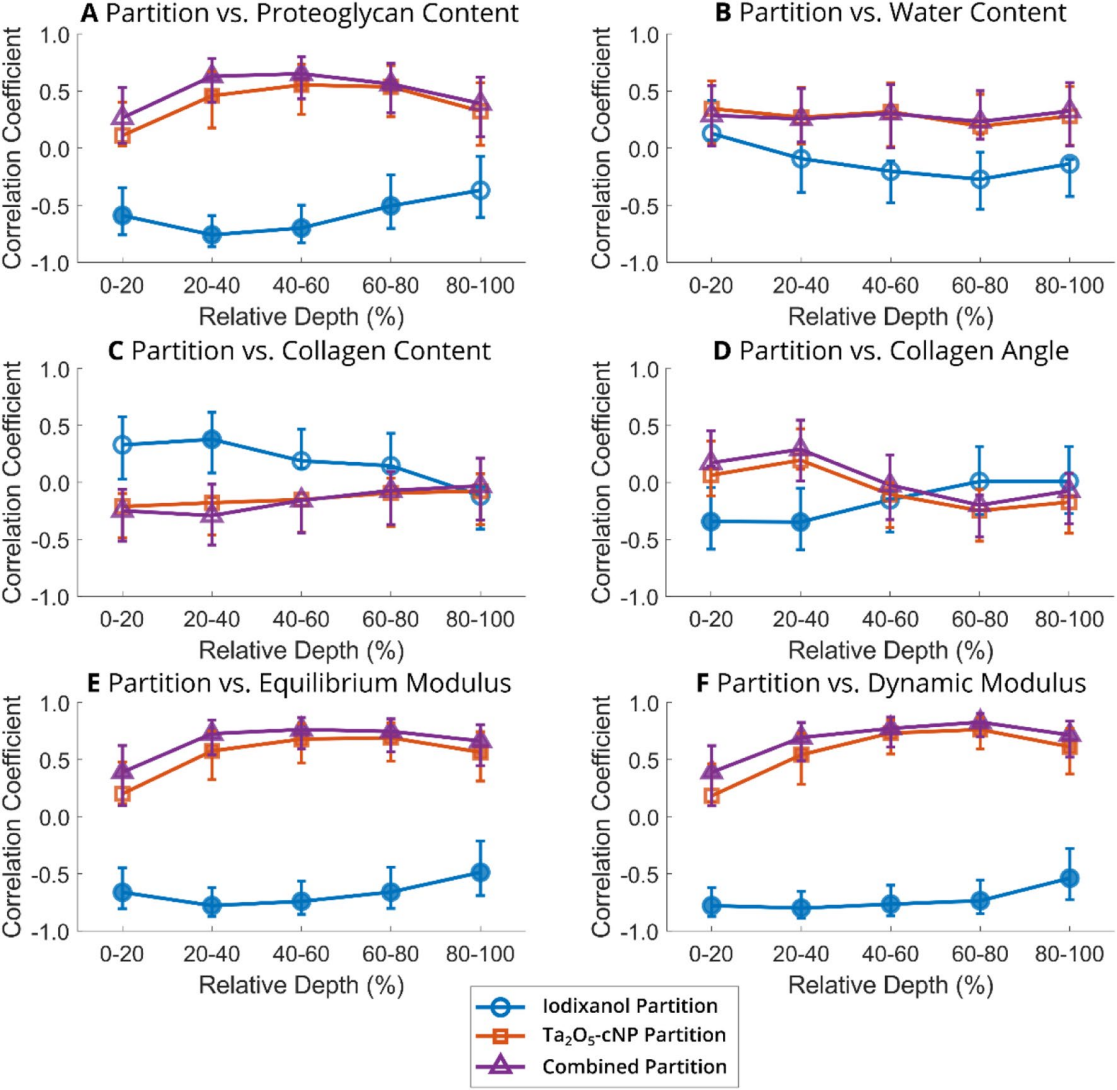
Spearman’s correlation coefficients between contrast agent partitions and reference methods as a function of depth at 96-hour timepoint. Contrast agent partition values at 96-hour timepoint and structure of articular cartilage (**A–D**) were calculated in 20% intervals, but function (**E–F**) of the articular cartilage was based on single value that was compared to contrast agent partitions in 20% intervals. Filled markers indicate significant correlation (*p* < 0.05) and the vertical bars the 95% confidence interval of the correlation coefficient. The articular cartilage surface is at 0% and the cartilage-bone interface at 100%

Upon conducting depthwise analysis, it became evident that at the 96-hour timepoint, the iodixanol partition exhibited a negative correlation with proteoglycan content at depths ranging from 0 to 80% of the cartilage (Fig. 4A), whereas a significant correlation with collagen content was only evident at depths of 20–40% (Fig. 4C) and with collagen angle only at the middle zone ranging from 0% to 40% (Fig. 4D). Conversely, the depthwise correlations of Ta_2_O_5_-cNP and combined partition with proteoglycan content were significant throughout the cartilage, except for the superficial layer (*i.e.*, 0–20%) (Fig. 4A). No depthwise correlation was observed between Ta_2_O_5_-cNP partition and collagen angle.

#### Contrast Agents’ Correlations with Articular Cartilage Function

Iodixanol partition strongly correlated with cartilage functional parameters, *i.e., E*_Eq_, *E*_Dyn_, *θ*, *α*, and *β*, especially at earlier timepoints, outperforming the Ta_2_O_5_-cNP and combined partitions (Table 1). The combined partition consistently displayed higher correlation coefficients compared to the Ta_2_O_5_-cNP partition. Significant correlations between iodixanol partition and cartilage function parameters began to emerge at the 3.5-hour timepoint and persisted in all diffusion timepoints (Table 1). A robust positive correlation existed with *θ*, and negative correlations with *E*_Eq_ and *E*_Dyn_ starting at the 3.5-hour timepoint. Correlation of the iodixanol partition with stress-relaxation time constant *α* (expressing permeability and porosity, Eq. 2) was strong at early timepoints, whereas with the stretching parameter *β* (expressing solid content, Eq. 2), the correlation was stronger after the 6-hour timepoint (Table 1).

Depthwise, the correlations between Ta_2_O_5_-cNP partition and function parameters were relatively similar between 20 and 100%, while in the superficial zone (0–20%), the correlations were not significant (Fig. 4E, F). The combined partition improved the correlations, and significant correlations were present in all cartilage layers compared to the Ta_2_O_5_-cNP partition. Iodixanol partition strongly and negatively correlated with *E*_Eq_ and *E*_Dyn_, and in some cases, the absolute correlation coefficient was higher than combined partition. Compared to the combined partition, iodixanol partition showed a more constant correlation throughout the cartilage and the lowest correlations were found at 80–100% depth and not in the superficial layer (Fig. 4E, F).

#### Distinction Between Healthy and Early-Stage OA Samples

**Fig. 5:**
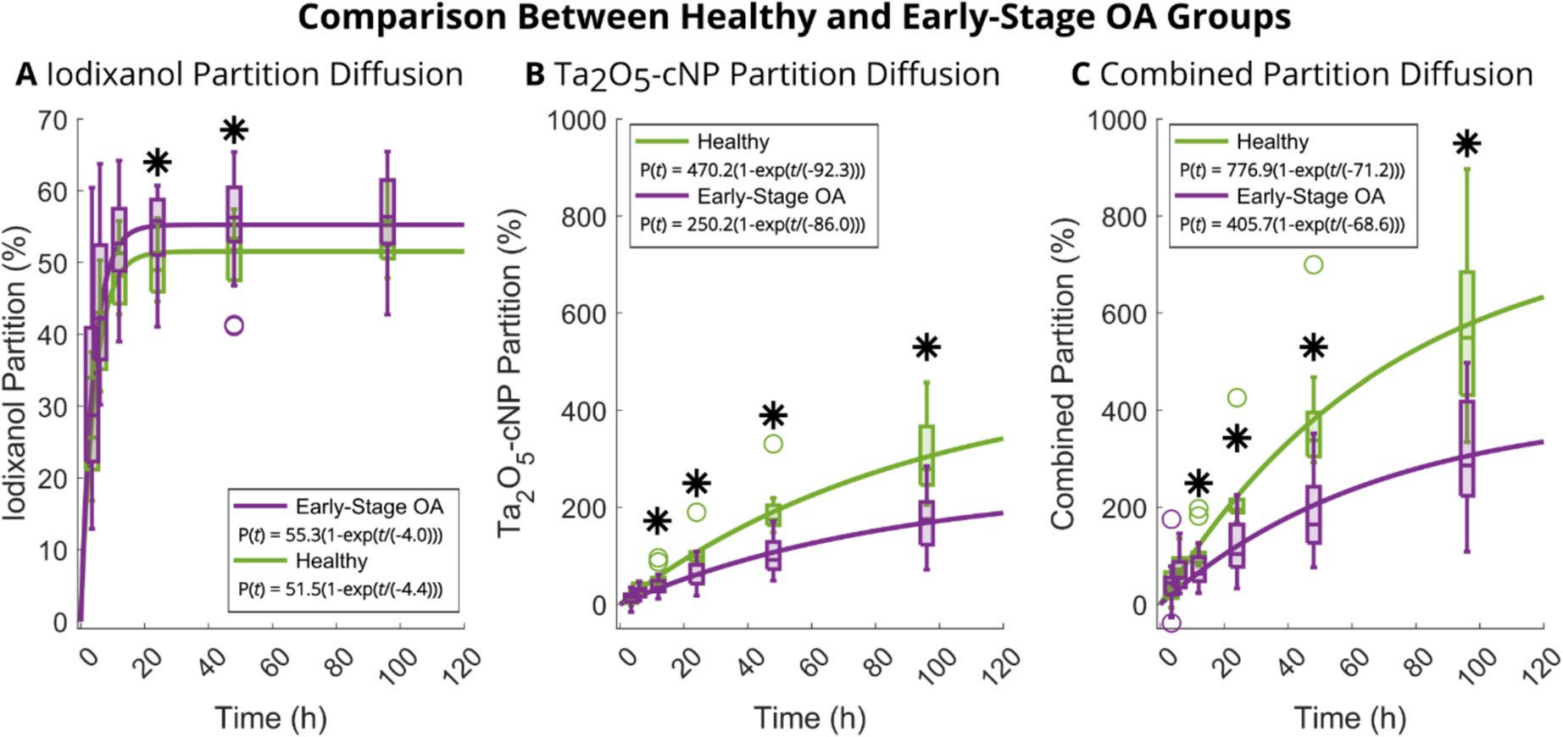
Comparison between healthy and early-stage osteoarthritis (OA) groups of Part A. Diffusion of contrast agent partition as a function of time of iodixanol (**A**), cationic tantalum oxide nanoparticle (Ta_2_O_5_-cNP) (**B**), and combined partition (**C**) (*i.e.*, Ta_2_O_5_-cNP partition divided by iodixanol partition) in healthy (Mankin score < 1.5, *n* = 10) and early-stage osteoarthritis (Mankin score 1.5–4, *n* = 20) samples. The fits were calculated by averaging the measured points at each timepoint and then using Eq. 1. The boxes indicate 75th percentile, the whiskers indicate 95th percentile, and circles indicate outliers that are more than 1.5 times away from interquartile range of the box. Asterisks mark the significant differences (*p* < 0.05) between the healthy and early-stage OA sample groups

The diffusion of iodixanol into cartilage samples in both healthy and early-stage OA groups was swifter compared to Ta_2_O_5_-cNP, reaching diffusion equilibrium at around 24 hours (Fig. 5A). Conversely, Ta_2_O_5_-cNP did not reach diffusion equilibrium during the bath immersion period (Fig. 5B). The *τ* for iodixanol, Ta_2_O_5_-cNP, and the combined partition did not display a significant difference between healthy and early-stage OA sample groups (Fig. 5). However, a notable finding emerged regarding *P*_Max_ values. Specifically, the *P*_Max_ of iodixanol was significantly lower in healthy cartilage compared to early-stage OA cartilage (Fig. 5A) but not at the 96-hour timepoint (Table 2). In contrast, both Ta_2_O_5_-cNP and the combined partition exhibited significantly higher *P*_Max_ and *P*_96h_ values in healthy cartilage compared to degenerated cartilage (Fig. 5B, C and Table 2).

**Table 2:**
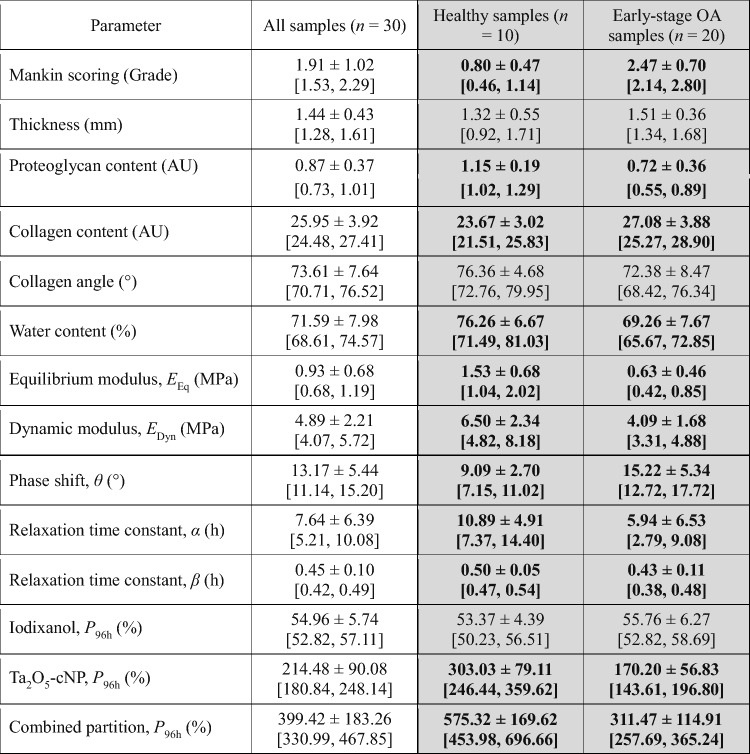
Properties of healthy and early-stage osteoarthritis (OA) samples in Part A

The groups were formed based on the Mankin scoring (the first row): the healthy group had grades between 0 and 1.5 and the early-stage OA group had grades between 1.5 and 4. For each parameter mean and standard deviation values are given in the first row. The lower and upper 95% confidence interval values are given in the brackets in the second row. Statistically significant difference (*p* < 0.05) between healthy and early-stage OA sample groups (columns with gray background) are indicated with bold typeface. AU = arbitrary unit. *P*_96h_ = contrast agent partition at 96-hour timepoint

The depth-related differences between healthy and early-stage OA cartilage were also examined (Fig. 3C and D), and no significant differences were detected with iodixanol at any depth or timepoint, but significant differences were observed starting from 12-hour timepoint at relative depth of 0–56% and 0–55% with Ta_2_O_5_-cNP and combined partition, respectively. A similar trend (*i.e.*, the superficial half of the cartilage has significant differences between the Mankin groups) continued at the following timepoints (not shown in Fig. 3D), until at 96-hour timepoint, where the significant differences were detected between the relative depths of 0–84% and 0–81% with Ta_2_O_5_-cNP and combined partition, respectively.

Significant differences were observed between the groups for almost all reference parameters (Table 2). Only thickness and collagen angle did not have significant differences between the groups. When assessing the sensitivity of contrast agents, the first significant difference in contrast agent partition was observed at the 12-hour timepoint for Ta_2_O_5_-cNP and combined partition, and at the 24-hour timepoint for iodixanol, with differences increasing over time (Fig. 5). However, iodixanol did not have significant difference between the groups at 96-hour timepoint.

### Part B: Diffusion Dynamics Between the Contrast Agents

#### *Interplay Between Ta*_*2*_*O*_*5*_*-cNP and Iodixanol and Impact on Diffusion Rates*

The diffusion of iodixanol and Ta_2_O_5_-cNP together and alone was observed and iodixanol affected Ta_2_O_5_-cNP diffusion, but not vice versa. When iodixanol was introduced first, Ta_2_O_5_-cNP diffusion significantly slowed (*τ* increased from 68.3 hours to 774.4 hours, and the reached partition at the 144-hour timepoint decreased from 890.2% to 370.7%). However, iodixanol diffusion remained relatively consistent even after Ta_2_O_5_-cNP introduction (*τ* was 5.2 hours and 8.9 hours before and after Ta_2_O_5_-cNP diffusion, respectively, with corresponding *P*_Max_ values of 62.9% and 77.2%).

## Discussion

In this study, we utilized an experimental PCD-CT setup with a dual-contrast agent method for CECT imaging of *ex vivo* equine articular cartilage explants. Our results demonstrate that the combination of nanoparticle- and molecule-based contrast agents effectively assesses cartilage structure and function: iodixanol reflects cartilage porosity and the collagen network, while Ta_2_O_5_-cNP reveals solid content, mainly proteoglycans. Therefore, the dual-contrast agent method could detect early-stage OA in cartilage and is applicable to clinical CT scanners, which inherently have larger voxel sizes. Since our analysis focused on bulk cartilage, the method remains robust even at lower spatial resolutions. These results represent a promising advancement for contrast-enhanced cartilage analysis and improve the dual-contrast agent method compared to a combination of two molecular agents, such as cationic iodinated CA4+ and non-ionic gadolinium-based gadoteridol.

The accuracy of the concentration estimation (*i.e.*, error in validation) using the iodine-tantalum combination is comparable to that of the iodine-gadolinium combination used in earlier studies [27, 37, 39–41, 51, 52]. However, the non-ionic iodixanol used in this study shows significant correlations with multiple reference parameters (Table 1), whereas non-ionic gadoteridol showed poor or insignificant correlations as reported in previous studies [27, 37, 39–41, 51, 52]. Given that non-ionic molecular contrast agents are neutral in charge and similar in size, and that there is no clear evidence suggesting difference in diffusion between non-ionic contrast agents in cartilage [21], the observed differences in correlations likely result from more accurate concentration estimation specific to cartilage. This finding suggests that the iodine-tantalum combination provides more robust material decomposition, with reduced susceptibility to imaging artifacts, such as beam hardening. This robustness likely stems from the wider *K*-edge separation between iodine and tantalum (33.2 keV and 67.4 keV, respectively) compared to iodine and gadolinium (33.2 keV and 50.2 keV, respectively), which allows for a better balance between low and high energy bins, and, potentially, improves the spectral separation of the contrast agents, especially when combined with PCD-CT.

In Part A of this study, iodixanol partition reflects collagen network’s effect on cartilage porosity (Table 1). A more organized matrix, represented by collagen angle [53], allows less free water (and iodixanol) inside, leading to iodixanol partition’s significant correlation with collagen angle, collagen content, and dynamic modulus *E*_Dyn_ (Table 1). This result suggests that iodixanol partition indicates collagen organization, as also reported by Tuppurainen et al. [54]. Similarly, the robust positive correlation of iodixanol partition with *θ* is attributed to the collagen network (Table 1). Degeneration leads to increased viscosity, as expressed by higher *θ* (Table 2), due to alterations in the collagen network architecture [55]. The structure-diffusion relationship further highlights that stress-relaxation time coefficient *α* (which reflects cartilage porosity) correlates negatively with iodixanol partition (Table 1). Therefore, lower porosity leads to lower iodixanol partitions and slower diffusion, underscoring the connection between iodixanol and cartilage porosity.

As the cationic Ta_2_O_5_-cNPs bind electrostatically to the anionic proteoglycans within cartilage [24, 43], and that the proteoglycans substantially contribute to cartilage equilibrium stiffness [56, 57], the Ta_2_O_5_-cNP partition strongly and positively correlates with the equilibrium modulus, *E*_Eq_ (Table 1). The proteoglycan content and distribution as well as the equilibrium modulus reflect cartilage health; thus, the Ta_2_O_5_-cNP partition also negatively correlates with Mankin scoring (Table 1), similarly as reported by Jäntti et al. [43], and a significant difference in *P*_Max_ of Ta_2_O_5_-cNPs exists between healthy and early-stage OA cartilage (Fig. 5B). The Ta_2_O_5_-cNP partition positively correlates with *β* (reflecting cartilage’s solid content, *i.e.*, the higher the *β*, the higher the solid content), notably from the 24-hour timepoint onward (Table 1). Therefore, the cartilage viscosity (*θ*) or porosity (*α*) does not influence Ta_2_O_5_-cNPs’ diffusion, as is the case for iodixanol, but rather by solid content (particularly proteoglycans) as previously discussed. This finding is in line with that reported by Jäntti et al. [43] and Tuppurainen et al. [54]. However, Jäntti et al. [43] also reported a significant correlation between Ta_2_O_5_-cNPs and *α* with a single-contrast agent method indicating that Ta_2_O_5_-cNPs alone are affected by porosity. Thus, the interactions between the nanoparticles and contrast agents in this study can alter the behavior of the contrast agents. Because the Ta_2_O_5_-cNP partition serves as the numerator in the calculation of the combined partition, the correlations from the combined partition reflect those of the Ta_2_O_5_-cNP partition. However, when compared to Ta_2_O_5_-cNP partition alone, the combined partition strengthens correlations (*e.g.*, with proteoglycan content, collagen angle, *E*_Eq_, *θ*, and *α*, Table 1) and improves them (*e.g.*, with *E*_Dyn_ and *β*, Table 1), which illustrates the enhanced sensitivity enabled by the combination of nanoparticle and molecular contrast agents.

In Part A, we divided the samples into groups based on Mankin scoring, to better resolve the impact of early-stage OA on the various parameters. We also examined the dependence of cartilage plug location within the tissue, and all other reference parameters had significant differences except collagen content (Supplementary Table 1). This result was expected as Fugazzola et al. [46] had shown that most of the structural and functional parameters have strong site specificity. The differences between healthy (Mankin grades: < 1.5) and early-stage OA (Mankin grades: 1.5–4) samples are evident (see Supplementary Figure 1) as determined using reference methods. Some superficial damage is present in the early-stage OA sample group with an average Mankin grade of 2.47. In addition, these samples possess on average 37.4% lower proteoglycan content compared to the healthy group, indicating relatively mild degeneration. The total water content of early-stage OA samples is lower, even though elevated water content is typically associated with OA. This result likely manifests due to the lowered proteoglycan content and the fact that, since OA is in its early stage, the free water content of the cartilage is not yet significantly increased. In addition, the similar *τ* of iodixanol, Ta_2_O_5_-cNP, and combined partition between healthy and early-stage OA cartilage samples (Fig. 5) indicate that deterioration does not significantly impact the diffusion speed of the contrast agents. Iodixanol partition shows a significant difference between healthy and early-stage OA groups at the 24-hour and 48-hour timepoints, but not at the 96-hour timepoint. However, Ta_2_O_5_-cNP and combined partition reflect significant differences between the groups already at the 12-hour timepoint. The combined partition also negatively correlates with *θ* and positively with *α* and *β*, beginning at the 12-hour timepoint (Table 1). Particularly, the strong correlation with *β* indicates a significant influence of solid content on the combined partition. These findings align with indicators of early-stage OA, highlighting the potential of our approach for early detection of OA.

**Fig. 6:**
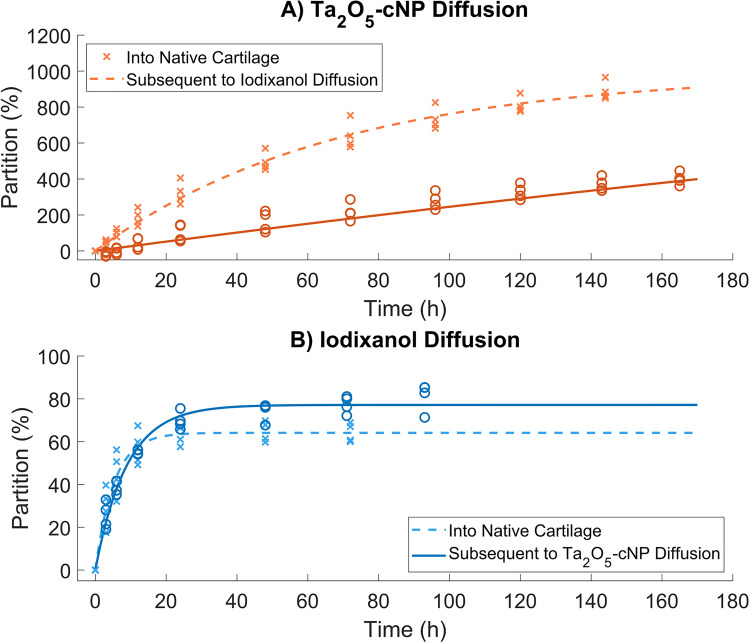
Contrast agent diffusion into native cartilage and subsequent to the other contrast agent. Part B of the study involved tracking contrast agent partition over time to examine how the contrast agents interacted with each other. In subfigure **A**, the diffusion of cationic tantalum oxide nanoparticles (Ta_2_O_5_-cNPs) is followed into native cartilage and into cartilage subsequent to iodixanol diffusion (*n* = 4). Similarly, in subfigure **B**, the diffusion of iodixanol into native cartilage, and into cartilage subsequent to Ta_2_O_5_-cNP diffusion (*n* = 4) is followed

Since combining nanoparticle- and molecule-based contrast agents for diffusion into articular cartilage is a novel concept in cartilage imaging, we also report on the interactions between the two contrast agents in Part B of this study. Iodixanol lowers the maximum partition of Ta_2_O_5_-cNPs to about 50% of that without iodixanol (Fig. 6A), whereas Ta_2_O_5_-cNPs do not affect the diffusion of iodixanol (Fig. 6B). We attribute this effect to the difference in size between iodixanol molecules (sand-like) and Ta_2_O_5_-cNPs (pebble-like). To draw a simplistic analogy, when a container is initially filled with pebbles (Ta_2_O_5_-cNPs), there is still space for sand (iodixanol), but the reverse arrangement does not allow the pebbles to fit in, and they accumulate on top of the sand. In our case, however, iodixanol does not fully impede the Ta_2_O_5_-cNP diffusion but rather leads to reduced accumulation. We noted a tenfold increase in the *τ* of Ta_2_O_5_-cNPs in Part B with iodixanol compared to without iodixanol. However, since this was not observed in the Part A, where the *τ* had similar values as in Part B without iodixanol, we suspect that small sample size in Part B affords poor fitting of the Ta_2_O_5_-cNPs curve when iodixanol is present, and thus poor *τ* values. The iodixanol diffusion also increases in the presence of Ta_2_O_5_-cNPs (Fig. 6B), but we speculate that this result may be due to the small sample size of four and potential errors in concentration estimation, as a consequence of using different material decomposition methods (*i.e.*, decomposition to one contrast agent vs. two) rather than reflecting its true behavior. We do not suspect that the different voxel sizes between Part A and B introduced significant errors since the voxel size was relatively small compared to the cartilage thickness. With an average thickness of 1.44 mm, the PCD-CT had 21 pixels and the EID-CT had an average of 57 pixels in the depth direction. Also, in the main analysis of Part A and B, the depthwise cartilage data were averaged to a single bulk value. This result indicates that the method is applicable to CT scanners with larger voxel sizes, such as clinical CT scanners, as long the bulk cartilage can be distinguished in sufficient detail.

This study has limitations that need to be acknowledged. Firstly, the scanning duration in our study was relatively long (*i.e.*, approx. 13 minutes plus the handling time) primarily due to our CT setups. Consequently, an error arose in the diffusion analysis since the contrast agents continued to diffuse inside cartilage even though the sample was not in the contrast agent bath. However, this error is a systematic error and affects all the samples and contrast agents similarly. Secondly, the Ta_2_O_5_-cNPs never reached diffusion equilibrium during immersion as evident from the diffusion data (Fig. 5) and the depthwise profiles (Fig. 3). As a result, errors were introduced into the estimated equilibrium partition *P*_Max_ and diffusion time coefficient *τ* values for Ta_2_O_5_-cNP and combined partition data and made the correlations not significant, thus the 96-hour timepoint was used to represent the late diffusion (*e.g.*, in Fig. 4 and Table 2). Thirdly, the slow diffusion limits the clinical viability of Ta_2_O_5_-cNP even if iodixanol improved the correlation. In future studies, we will examine approaches to modify the nanoparticles, such as altering their size or charge, to accelerate diffusion. However, the introduced dual-contrast agent CT method remains applicable as a spectral microscopy method or 3D histological tool. Lastly, the statistical analysis has limitations, as the low number of observations did not allow advanced statistical models for the analysis, only simple tests and correlations were applied. However, the use of the methods was justified by the questions they needed to answer.

In conclusion, PCD-CT successfully distinguishes between nanoparticle and molecular contrast agents in *ex vivo* articular cartilage samples with a single scan. As hypothesized, PCD-CT combined with the dual-contrast agent method enables quantitative assessment and differentiation of healthy and early-stage OA cartilage after 12 hours of immersion. Ta_2_O_5_-cNPs show sensitivity to structure-related parameters beyond proteoglycans, especially when the partition of Ta_2_O_5_-cNPs is compared to that of non-ionic iodixanol (*i.e.*, combined partition). We also hypothesized that the presence of one contrast agent would alter the diffusion of the other, and indeed, the Ta_2_O_5_-cNP partition approximately halved in the presence of iodixanol. A key benefit is the size difference between the nanoparticle and molecular agents, allowing for a more comprehensive assessment of cartilage properties. Additionally, the favorable *K*-edge energy separation between iodine and tantalum (compared to iodine and gadolinium) improves concentration estimation with PCD-CT. Notably, these advantages are achieved in a single scan, eliminating the need for multiple acquisitions and alignment of images. Future studies will explore the use of this spectral dual-contrast CECT technique in OA models to better understand interactions between nanoparticle and molecular contrast agents, address tissue-related acquisition artifacts, and evaluate the safety of dual-contrast agents. These findings lay the groundwork for developing this technique as a quantitative tool for articular cartilage imaging and potentially for broader applications in imaging scenarios requiring enhanced contrast and precision.

## Supplementary Information

Below is the link to the electronic supplementary material.

Supplementary file1 (PDF 644 kb)

## References

[1] Taguchi, K. Energy-sensitive photon counting detector-based X-ray computed tomography. *Radiol. Phys. Technol.* 10:8–22, 2017. 10.1007/s12194-017-0390-9. 28138947 10.1007/s12194-017-0390-9

[2] Willemink, M. J., M. Persson, A. Pourmorteza, N. J. Pelc, and D. Fleischmann. Photon-counting CT: technical principles and clinical prospects. *Radiology*. 289:293–312, 2018. 10.1148/radiol.2018172656. 30179101 10.1148/radiol.2018172656

[3] Leng, S., M. Bruesewitz, S. Tao, K. Rajendran, A. F. Halaweish, N. G. Campeau, et al. Photon-counting detector CT: system design and clinical applications of an emerging technology. *Radiographics*. 39:729–743, 2019. 10.1148/rg.2019180115. 31059394 10.1148/rg.2019180115 PMC6542627

[4] Flohr, T., M. Petersilka, A. Henning, S. Ulzheimer, J. Ferda, and B. Schmidt. Photon-counting CT review. *Phys. Med.* 79:126–136, 2020. 10.1016/j.ejmp.2020.10.030. 33249223 10.1016/j.ejmp.2020.10.030

[5] Danielsson, M., M. Persson, and M. Sjölin. Photon-counting x-ray detectors for CT. *Phys. Med. Biol.* 2021. 10.1088/1361-6560/abc5a5. 33113525 10.1088/1361-6560/abc5a5

[6] Hsieh, S. S., S. Leng, K. Rajendran, S. Tao, and C. H. McCollough. Photon counting CT: clinical applications and future developments. *IEEE Trans. Radiat. Plasma Med. Sci.* 5:441–452, 2021. 10.1109/TRPMS.2020.3020212. 34485784 10.1109/trpms.2020.3020212 PMC8409241

[7] Tortora, M., L. Gemini, I. D’Iglio, L. Ugga, G. Spadarella, and R. Cuocolo. Spectral photon-counting computed tomography: a review on technical principles and clinical applications. *J. Imaging*. 8:112, 2022. 10.3390/jimaging8040112. 35448239 10.3390/jimaging8040112 PMC9029331

[8] McCollough, C. H., K. Rajendran, S. Leng, L. Yu, J. G. Fletcher, K. Stierstorfer, et al. The technical development of photon-counting detector CT. *Eur. Radiol.* 33:5321–5330, 2023. 10.1007/s00330-023-09545-9. 37014409 10.1007/s00330-023-09545-9 PMC10330290

[9] McCollough, C. H., S. Leng, L. Yu, and J. G. Fletcher. Dual- and multi-energy CT: principles, technical approaches, and clinical applications. *Radiology*. 276:637–653, 2015. 10.1148/radiol.2015142631. 26302388 10.1148/radiol.2015142631 PMC4557396

[10] Chellini, D., and K. Kinman. Dual-energy CT principles and applications. *Radiol. Technol.* 91:561CT-576CT, 2020. 32606242

[11] Tatsugami, F., T. Higaki, Y. Nakamura, Y. Honda, and K. Awai. Dual-energy CT: minimal essentials for radiologists. *Jpn. J. Radiol.* 40:547–559, 2022. 10.1007/s11604-021-01233-2. 34981319 10.1007/s11604-021-01233-2 PMC9162973

[12] Esquivel, A., A. Ferrero, A. Mileto, F. Baffour, K. Horst, P. S. Rajiah, et al. Photon-counting detector CT: key points radiologists should know. *Korean J. Radiol.* 23:854–865, 2022. 10.3348/kjr.2022.0377. 36047540 10.3348/kjr.2022.0377 PMC9434736

[13] Gutjahr, R., A. F. Halaweish, Z. Yu, S. Leng, L. Yu, Z. Li, et al. Human imaging with photon counting-based computed tomography at clinical dose levels. *Invest. Radiol.* 51:421–429, 2016. 10.1097/RLI.0000000000000251. 26818529 10.1097/RLI.0000000000000251 PMC4899181

[14] Allen, K. D., L. M. Thoma, and Y. M. Golightly. Epidemiology of osteoarthritis. *Osteoarthr. Cartil.* 30:184–195, 2022. 10.1016/j.joca.2021.04.020. 10.1016/j.joca.2021.04.020 PMC10735233 34534661

[15] Sakellariou, G., P. G. Conaghan, W. Zhang, J. W. J. Bijlsma, P. Boyesen, M. A. D’Agostino, et al. EULAR recommendations for the use of imaging in the clinical management of peripheral joint osteoarthritis. *Ann. Rheum. Dis.* 76:1484–1494, 2017. 10.1136/annrheumdis-2016-210815. 28389554 10.1136/annrheumdis-2016-210815

[16] Hunter, D. J., and S. Bierma-Zeinstra. Osteoarthritis. *Lancet*. 393:1745–1759, 2019. 10.1016/S0140-6736(19)30417-9. 31034380 10.1016/S0140-6736(19)30417-9

[17] Mahmoudian, A., L. S. Lohmander, A. Mobasheri, M. Englund, and F. P. Luyten. Early-stage symptomatic osteoarthritis of the knee—time for action. *Nat. Rev. Rheumatol.* 17:621–632, 2021. 10.1038/s41584-021-00673-4. 34465902 10.1038/s41584-021-00673-4

[18] Armstrong, C. G., and V. C. Mow. Variations in the intrinsic mechanical properties of human articular cartilage with age, degeneration, and water content. *J. Bone Joint Surg.* 64:88–94, 1982. 10.2106/00004623-198264010-00013. 7054208

[19] Nieminen, H. J., T. Ylitalo, S. Karhula, J.-P. Suuronen, S. Kauppinen, R. Serimaa, et al. Determining collagen distribution in articular cartilage using contrast-enhanced micro-computed tomography. *Osteoarthr. Cartil.* 23:1613–1621, 2015. 10.1016/j.joca.2015.05.004. 10.1016/j.joca.2015.05.004 PMC4565718 26003951

[20] Pouran, B., V. Arbabi, A. A. Zadpoor, and H. Weinans. Isolated effects of external bath osmolality, solute concentration, and electrical charge on solute transport across articular cartilage. *Med. Eng. Phys.* 38:1399–1407, 2016. 10.1016/j.medengphy.2016.09.003. 27720635 10.1016/j.medengphy.2016.09.003

[21] Ter Voert, C. E. M., R. Y. N. Kour, B. C. J. van Teeffelen, N. Ansari, and K. S. Stok. Contrast-enhanced micro-computed tomography of articular cartilage morphology with ioversol and iomeprol. *J. Anat.* 237:1062–1071, 2020. 10.1111/joa.13271. 32683740 10.1111/joa.13271 PMC7704241

[22] Boos, M. A., M. W. Grinstaff, S. R. Lamandé, and K. S. Stok. Contrast-enhanced micro-computed tomography for 3D visualization and quantification of glycosaminoglycans in different cartilage types. *Cartilage*. 13:486S-494S, 2021. 10.1177/19476035211053820. 34696603 10.1177/19476035211053820 PMC8804852

[23] Nelson, B. B., R. C. Stewart, C. E. Kawcak, J. D. Freedman, A. N. Patwa, B. D. Snyder, et al. Quantitative evaluation of equine articular cartilage using cationic contrast-enhanced computed tomography. *Cartilage*. 12:211–221, 2021. 10.1177/1947603518812562. 33722083 10.1177/1947603518812562 PMC7970376

[24] Lawson, T., A. Joenathan, A. Patwa, B. D. Snyder, and M. W. Grinstaff. Tantalum oxide nanoparticles for the quantitative contrast-enhanced computed tomography of ex vivo human cartilage: assessment of biochemical composition and biomechanics. *ACS Nano*. 15:19175–19184, 2021. 10.1021/acsnano.1c03375. 34882411 10.1021/acsnano.1c03375

[25] Saukko, A. E. A., J. T. J. Honkanen, W. Xu, S. P. Väänänen, J. S. Jurvelin, V.-P. Lehto, et al. Dual contrast CT method enables diagnostics of cartilage injuries and degeneration using a single CT image. *Ann. Biomed. Eng.* 45:2857–2866, 2017. 10.1007/s10439-017-1916-3. 28924827 10.1007/s10439-017-1916-3

[26] Myller, K. A. H., M. J. Turunen, J. T. J. Honkanen, S. P. Väänänen, J. T. Iivarinen, J. Salo, et al. In vivo contrast-enhanced cone beam CT provides quantitative information on articular cartilage and subchondral bone. *Ann. Biomed. Eng.* 45:811–818, 2017. 10.1007/s10439-016-1730-3. 27646147 10.1007/s10439-016-1730-3

[27] Honkanen, M. K. M. M., H. Matikka, J. T. J. J. Honkanen, A. Bhattarai, M. W. Grinstaff, A. Joukainen, et al. Imaging of proteoglycan and water contents in human articular cartilage with full-body CT using dual contrast technique. *J. Orthop. Res.* 37:1059–1070, 2019. 10.1002/jor.24256. 30816584 10.1002/jor.24256 PMC6594070

[28] Honkanen, M. K. M., A. E. A. Saukko, M. J. Turunen, R. Shaikh, M. Prakash, G. Lovric, et al. Synchrotron MicroCT reveals the potential of the dual contrast technique for quantitative assessment of human articular cartilage composition. *J. Orthop. Res.* 38:563–573, 2020. 10.1002/jor.24479. 31535728 10.1002/jor.24479 PMC7065106

[29] Hrvoje, L., M. W. Greenstaff, H. Lusic, and M. W. Grinstaff. X-ray-computed tomography contrast agents. *Chem. Rev.* 113:1641–1666, 2014. 10.1021/cr200358s. 10.1021/cr200358s PMC3878741 23210836

[30] Joshi, N. S., P. N. Bansal, R. C. Stewart, B. D. Snyder, and M. W. Grinstaff. Effect of contrast agent charge on visualization of articular cartilage using computed tomography: exploiting electrostatic interactions for improved sensitivity. *J. Am. Chem. Soc.* 131:13234–13235, 2009. 10.1021/ja9053306. 19754183 10.1021/ja9053306

[31] Palmer, A. W., R. E. Guldberg, and M. E. Levenston. Analysis of cartilage matrix fixed charge density and three-dimensional morphology via contrast-enhanced microcomputed tomography. *Proc. Natl. Acad. Sci. USA*. 103:19255–19260, 2006. 10.1073/pnas.0606406103. 17158799 10.1073/pnas.0606406103 PMC1748213

[32] Freedman, J. D., H. Lusic, B. D. Snyder, and M. W. Grinstaff. Tantalum oxide nanoparticles for the imaging of articular cartilage using X-ray computed tomography: visualization of ex vivo/in vivo murine tibia and ex vivo human index finger cartilage. *Angew. Chem. Int. Ed. Engl.* 53:8406–8410, 2014. 10.1002/anie.201404519. 24981730 10.1002/anie.201404519 PMC4303344

[33] Kim, J., D. Bar-Ness, S. Si-Mohamed, P. Coulon, I. Blevis, P. Douek, et al. Assessment of candidate elements for development of spectral photon-counting CT specific contrast agents. *Sci. Rep.* 8:12119, 2018. 10.1038/s41598-018-30570-y. 30108247 10.1038/s41598-018-30570-y PMC6092324

[34] Wei, B., X. Zhang, C. Zhang, Y. Jiang, Y.-Y. Fu, C. Yu, et al. Facile synthesis of uniform-sized bismuth nanoparticles for CT visualization of gastrointestinal tract in vivo. *ACS Appl. Mater. Interfaces*. 8:12720–12726, 2016. 10.1021/acsami.6b03640. 27144639 10.1021/acsami.6b03640

[35] Cole, L. E., R. D. Ross, J. M. Tilley, T. Vargo-Gogola, and R. K. Roeder. Gold nanoparticles as contrast agents in x-ray imaging and computed tomography. *Nanomedicine (Lond.)*. 10:321–341, 2015. 10.2217/nnm.14.171. 25600973 10.2217/nnm.14.171

[36] Si-Mohamed, S., D. P. Cormode, D. Bar-Ness, M. Sigovan, P. C. Naha, J.-B. Langlois, et al. Evaluation of spectral photon counting computed tomography K-edge imaging for determination of gold nanoparticle biodistribution in vivo. *Nanoscale*. 9:18246–18257, 2017. 10.1039/C7NR01153A. 28726968 10.1039/c7nr01153a PMC5709229

[37] Bhattarai, A., J. T. J. Honkanen, K. A. H. Myller, M. Prakash, M. Korhonen, A. E. A. Saukko, et al. Quantitative dual contrast CT technique for evaluation of articular cartilage properties. *Ann. Biomed. Eng.* 46:1038–1046, 2018. 10.1007/s10439-018-2013-y. 29654384 10.1007/s10439-018-2013-y

[38] Saukko, A. E. A., M. J. Turunen, M. K. M. Honkanen, G. Lovric, V. Tiitu, J. T. J. Honkanen, et al. Simultaneous quantitation of cationic and non-ionic contrast agents in articular cartilage using synchrotron MicroCT imaging. *Sci. Rep.* 9:7118, 2019. 10.1038/s41598-019-43276-6. 31068614 10.1038/s41598-019-43276-6 PMC6506503

[39] Bhattarai, A., B. Pouran, J. T. A. Mäkelä, R. Shaikh, M. K. M. Honkanen, M. Prakash, et al. Dual contrast in computed tomography allows earlier characterization of articular cartilage over single contrast. *J. Orthop. Res.* 38:2230–2238, 2020. 10.1002/jor.24774. 32525582 10.1002/jor.24774

[40] Paakkari, P., S. I. Inkinen, M. K. M. Honkanen, M. Prakash, R. Shaikh, M. T. Nieminen, et al. Quantitative dual contrast photon-counting computed tomography for assessment of articular cartilage health. *Sci. Rep.* 11:5556, 2021. 10.1038/s41598-021-84800-x. 33692379 10.1038/s41598-021-84800-x PMC7946949

[41] Bhattarai, A., J. T. A. Mäkelä, B. Pouran, H. Kröger, H. Weinans, M. W. Grinstaff, et al. Effects of human articular cartilage constituents on simultaneous diffusion of cationic and nonionic contrast agents. *J. Orthop. Res.* 39:771–779, 2021. 10.1002/jor.24824. 32767676 10.1002/jor.24824 PMC8048551

[42] Juntunen, M. A. K. K., S. I. Inkinen, J. H. Ketola, A. Kotiaho, M. Kauppinen, A. Winkler, et al. Framework for photon counting quantitative material decomposition. *IEEE Trans. Med. Imaging*. 39:35–47, 2020. 10.1109/TMI.2019.2914370. 31144630 10.1109/TMI.2019.2914370

[43] Jäntti, J., A. Joenathan, M. Fugazzola, J. Tuppurainen, J. T. J. Honkanen, J. Töyräs, et al. Cationic tantalum oxide nanoparticle contrast agent for micro computed tomography reveals articular cartilage proteoglycan distribution and collagen architecture alterations. *Osteoarthr. Cartil.* 32:299–309, 2024. 10.1016/j.joca.2023.11.020. 10.1016/j.joca.2023.11.020 38061579

[44] Kulmala, K. A. M., H. M. Karjalainen, H. T. Kokkonen, V. Tiitu, V. Kovanen, M. J. Lammi, et al. Diffusion of ionic and non-ionic contrast agents in articular cartilage with increased cross-linking–contribution of steric and electrostatic effects. *Med. Eng. Phys.* 35:1415–1420, 2013. 10.1016/j.medengphy.2013.03.010. 23622944 10.1016/j.medengphy.2013.03.010

[45] Bursac, P. M., L. E. Freed, R. J. Biron, and G. Vunjak-Novakovic. Mass transfer studies of tissue engineered cartilage. *Tissue Eng.* 2:141–150, 1996. 10.1089/ten.1996.2.141. 19877936 10.1089/ten.1996.2.141

[46] Fugazzola, M., M. T. Nissinen, J. Jäntti, J. Tuppurainen, S. Plomp, N. Te Moller, et al. Composition, architecture and biomechanical properties of articular cartilage in differently loaded areas of the equine stifle. *Equine Vet. J.* 2023. 10.1111/evj.13960. 37376723 10.1111/evj.13960

[47] June, R. K., S. Ly, and D. P. Fyhrie. Cartilage stress-relaxation proceeds slower at higher compressive strains. *Arch. Biochem. Biophys.* 483:75–80, 2009. 10.1016/j.abb.2008.11.029. 19111671 10.1016/j.abb.2008.11.029

[48] Hayes, W. C., L. M. Keer, G. Herrmann, and L. F. Mockros. A mathematical analysis for indentation tests of articular cartilage. *J. Biomech.* 5:541–551, 1972. 10.1016/0021-9290(72)90010-3. 4667277 10.1016/0021-9290(72)90010-3

[49] Kiviranta, P., J. Rieppo, R. K. Korhonen, P. Julkunen, J. Töyräs, and J. S. Jurvelin. Collagen network primarily controls Poisson’s ratio of bovine articular cartilage in compression. *J. Orthop. Res.* 24:690–699, 2006. 10.1002/jor.20107. 16514661 10.1002/jor.20107

[50] Mankin, H. J., and L. Lippiello. Biochemical and metabolic abnormalities in articular cartilage from osteo-arthritic human hips. *J. Bone Joint Surg. Am.* 52:424–434, 1970. 4246573

[51] Orava, H., P. Paakkari, J. Jäntti, M. K. M. Honkanen, J. T. J. Honkanen, T. Virén, et al. Triple contrast computed tomography reveals site-specific biomechanical differences in the human knee joint-a proof of concept study. *J. Orthop. Res.* 2023. 10.1002/jor.25683. 37593815 10.1002/jor.25683

[52] Saukko, A. E. A., O. Nykänen, J. K. Sarin, M. J. Nissi, N. C. R. Te Moller, H. Weinans, et al. Dual-contrast computed tomography enables detection of equine posttraumatic osteoarthritis in vitro. *J. Orthop. Res.* 40:703–711, 2022. 10.1002/jor.25066. 33982283 10.1002/jor.25066

[53] Ebrahimi, M., M. J. Turunen, M. A. Finnilä, A. Joukainen, H. Kröger, S. Saarakkala, et al. Structure-function relationships of healthy and osteoarthritic human tibial cartilage: experimental and numerical investigation. *Ann. Biomed. Eng.* 48:2887–2900, 2020. 10.1007/s10439-020-02559-0. 32648191 10.1007/s10439-020-02559-0 PMC7723942

[54] Tuppurainen, J., P. Paakkari, J. Jäntti, M. T. Nissinen, M. C. Fugazzola, R. van Weeren, et al. Revealing detailed cartilage function through nanoparticle diffusion imaging: a computed tomography & finite element study. *Ann. Biomed. Eng.* 52:2584–2595, 2024. 10.1007/s10439-024-03552-7. 39012563 10.1007/s10439-024-03552-7 PMC11329549

[55] Ebrahimi, M., A. Turkiewicz, M. A. J. Finnilä, S. Saarakkala, M. Englund, R. K. Korhonen, et al. Associations of human femoral condyle cartilage structure and composition with viscoelastic and constituent-specific material properties at different stages of osteoarthritis. *J. Biomech.* 145:111390, 2022. 10.1016/j.jbiomech.2022.111390. 36442429 10.1016/j.jbiomech.2022.111390

[56] Franz, T., E. M. Hasler, R. Hagg, C. Weiler, R. P. Jakob, and P. Mainil-Varlet. In situ compressive stiffness, biochemical composition, and structural integrity of articular cartilage of the human knee joint. *Osteoarthr. Cartil.* 9:582–592, 2001. 10.1053/joca.2001.0418. 10.1053/joca.2001.0418 11520173

[57] Korhonen, R. K., and J. S. Jurvelin. Compressive and tensile properties of articular cartilage in axial loading are modulated differently by osmotic environment. *Med. Eng. Phys.* 32:155–160, 2010. 10.1016/j.medengphy.2009.11.004. 19955010 10.1016/j.medengphy.2009.11.004

