# Supplementary material for: Dual-Contrast Agent with Nanoparticle and Molecular Components in Photon-Counting Computed Tomography: Assessing Articular Cartilage Health
Source: Ann Biomed Eng. 2025 Mar 28;53(6):1423–38. doi: 10.1007/s10439-025-03715-0 (PMC12075350; doi:10.1007/s10439-025-03715-0)
Supplement: Supplementary file 1 — Supplementary file1 (PDF 644 kb) [file 10439_2025_3715_MOESM1_ESM.pdf]

Supplementary Material

Dual-Contrast Agent with Nanoparticle and Molecular Components in Photon-Counting Computed Tomography: Assessing Articular Cartilage Health

Petri Paakkari[1,2,*], Satu I. Inkinen[3], Jiri Jäntti[1,2], Juuso Tuppurainen[1,2], Maria C. Fugazzola[4], Anisha Joenathan[5], Sampo Ylisiurua[6,7], Miika T. Nieminen[7], Heikki Kröger[8,9], Santtu Mikkonen[1,10], René van Weeren[4], Brian D. Snyder[11], Juha Töyräs[1,12,13], Miitu K.M. Honkanen[2], Hanna Matikka[2], Mark W. Grinstaff[5,†], Juuso T.J. Honkanen[14,†], Janne T.A. Mäkelä[1,2,†]

[1]Department of Technical Physics, University of Eastern Finland, Kuopio, Finland

[2]Diagnostic Imaging Center, Kuopio University Hospital, Kuopio, Finland

[3]Diagnostic Center, University of Helsinki and Helsinki University Hospital, Helsinki, Finland

[4]Department of Clinical Sciences, Faculty of Veterinary Medicine, Utrecht University, Utrecht, The Netherlands

[5]Departments of Biomedical Engineering, Chemistry and Medicine, Boston University, Boston, MA, United States

[6]Oulu University Hospital, Oulu, Finland

[7]Research Unit of Medical Imaging, Physics and Technology, University of Oulu, Oulu, Finland

[8]Department of Orthopaedics and Traumatology, Kuopio University Hospital, Kuopio, Finland

[9]Musculoskeletal Research Unit, University of Eastern Finland, Kuopio, Finland

[10]Department of Environmental and Biological Sciences, University of Eastern Finland, Kuopio, Finland

[11]Boston Children's Hospital, Boston, MA, United States

[12]School of Electrical Engineering and Computer Science, The University of Queensland, Brisbane, Australia

[13]Science Service Center, Kuopio University Hospital, Kuopio, Finland

[14]Radiotherapy Department, Center of Oncology, Kuopio University Hospital, Kuopio, Finland

† These authors contributed equally to this work.

* This author is the corresponding author.

Address correspondence: Petri Paakkari, Department of Technical Physics, University of Eastern Finland, 70211 Kuopio, Finland. E-mail address: petri.paakkari@uef.fi

Materials and Methods

PCD-CT Setup

The experimental PCD-CT setup consisted of two-bin PCD (XC-Flite FX15, Xcounter AB, Stockholm, Sweden), mini-focus X-ray source (IXS1203MF, VJ X-Ray, Bohemia, USA), and motorized rotator (NR360S, Thorlabs Inc., Newton, USA). The PCD had two rows of 12 tiles (size of 25.6 × 12.8 mm^2) and there was 100 µm tile gap between each tile, thus, the active area of the PCD was 51.3 × 154.7 mm^2. The detector pixel size was 100 × 100 μm^2 and each pixel had two 12-bit counters. Source-to-detector and object-to-source distances were 86.09 cm and 58.91 cm, respectively, thus, the magnification was 1.46. The X-ray source had a 50 µm focal spot with the applied voltage and current were set to 120 kVp and 0.25 mA, respectively. Imaging time was 12.5 minutes, rotation speed was set to 0.5 degrees per second, and frame rate was set to 2 frames per second. After data acquisition, frames exceeding 360 degrees were excluded. To improve the signal-to-noise ratio, two consecutive frames were averaged, resulting in 720 frames in total. For signal-to-equivalent thickness calibration correction method [1], projection images of increasing thicknesses of polymethyl methacrylate (PMMA) plates (0, 5.0, 10.0, 20.0, 35.0, 55.0, 75.0, and 89.0 mm) covering the detector area were acquired. Water tube was included in all PCD-CT acquisitions to convert attenuation values into Hounsfield units (HUs).

Since the material decomposition relies on variations in attenuation across different energy ranges, careful consideration was paid into selecting the X-ray spectrum and energy bins. Strong filtering (3.0 mm aluminum and 0.5 mm copper) was applied to eliminate low-energy photons. This step was essential because photons with energy lower than 33.2 keV (the *K*-edge of iodine) could artificially elevate the attenuation of tantalum compared to iodine, even though the low-energy bin should ideally be dominated by iodine. Additionally, one of the adjustable energy thresholds of the PCD was set at 10 keV to filter out electronic noise, and the other to 80 keV, close to corresponding the *K*-edge of tantalum. It is worth noting that the PCD experienced some electrical drift, leading to detected photons having higher energy readings than their actual values [2]. To account for this, the higher threshold was set at 80 keV, as opposed to 70 keV, which is closer to the tantalum *K*-edge (*i.e.*, 67.4 keV). This adjustment was made to compensate for the approximately +10 keV electrical drift observed in the vicinity of 70 keV. Therefore, the captured energy bins were high-energy (HE) 80-120 keV and total-energy (TE) 10-120 keV, while the low-energy (LE) bin (10-80 keV) was calculated offline.

PCD-CT Data Processing and Analysis

Raw projection data was preprocessed to correct PCD-related inhomogeneities and artifacts, *i.e.*, gaps between PCD tiles, scattering, beam hardening, and ring artifacts. The tile gap between the PCD tile elements was filled using linear interpolation and inhomogeneities were corrected with PMMA signal-to-equivalent thickness calibration correction. Pixel-wise transformation maps from detected photon counts to equivalent

thicknesses for each energy bin were calculated separately by fitting a monoexponential model to PMMA thickness measurements.

A Gaussian filter was used to smooth the projections before reconstructions were calculated using the Feldkamp-Davis-Kress method and ASTRA toolbox (ver. 1.8.3, Imec-Vision Lab, University of Antwerp, CWI, Amsterdam, Netherlands) [3,4]. The voxel size in the reconstruction was 68.4 × 68.4 × 68.4 μm^3. Areas of air and water (approximately 3 × 3 mm^2) were averaged from the image stacks to convert the reconstruction voxel values into HUs. Data processing and analysis was done using MATLAB (R2018b, MathWorks, Natick, MA, USA).

After the reconstructions, the calibration solutions were analyzed for the calibration-based material decomposition method as described earlier [5]. Average HU values for each concentration of contrast agent solutions were computed for each energy bin and a linear model was fitted to the data (Fig. 2A). Then the fitted calibration coefficients (*i.e.*, slope values) were validated against validation solutions of contrast agent mixtures (Fig. 2B) by computing the error between the estimated and known true concentrations. The concentration estimation was conducted for different energy bin combinations: low and high energy (LE&HE) bins, low and total energy (LE&TE) bins, and high and total energy (HE&TE) bins. LE&TE combination was chosen to be used in subsequent analysis due to exhibiting the smallest error in contrast agent concentration estimation (data not shown here). The estimated concentrations were transformed to contrast agent partitions by dividing the estimated concentration by the concentration of the original bath [5]. Additionally, combined partition was calculated by dividing the Ta_2O_5-cNP partition by the iodixanol partition.

Depthwise HU profiles were analyzed perpendicular to the cartilage surface. Each depthwise point in the profile was averaged over an area of 25 × 25 pixels (approximately 1.71 × 1.71 mm^2) from the center of the sample. To remove the air and bone from the profiles, those of native cartilage (*i.e.*, 0-hour timepoint) were manually cropped. Other timepoints (*i.e.*, between 3.5 – 96 hour timepoints) were cropped by locating the cartilage surface based on HU values and using the native cartilage profile length of that sample as reference for the cartilage thickness. As all the reconstructed energy bin image stacks were in same orientation, there was no need for co-registration and HU profiles from all energy bin stacks were extracted simultaneously. The corresponding HU values of native cartilage were subtracted from all the profiles of each sample to ensure that attenuation of the cartilage did not cause errors in the concentration estimation. Next, the cropped HU profiles were converted to two contrast agent concentration profiles by using material decomposition method and linearly interpolated to 100 points. Finally, concentration profiles were converted to partition profiles by dividing each point with the original bath concentration of the contrast agent.

Mechanical Testing

The cartilage thickness was measured using a conventional microCT (Nikon XT H 225, Nikon Metrology Europe, Leuven, Belgium) with a voxel size of 50 × 50 × 50 μm^3. The indentation (plane-ended cylindrical indenter, radius = 0.55 µm) protocol was the same as in our previous work [6,7]: four-step stress-relaxation

(step size: 4% of remaining cartilage thickness, ramp rate: 100% of the cartilage thickness per second, relaxation time between steps: 600 s) and a sinusoidal loading protocol (peak-to-peak strain amplitude: 4% of the cartilage thickness, sinusoidal cycles: 4, frequency: 1 Hz).

Microscopic and Spectroscopic Characterization of Cartilage Composition

Fourier-transform infrared spectroscopy, polarized light microscopy, and digital densitometry methods were used for determination of collagen and proteoglycan contents, collagen fiber network orientation, and parallelism index, similarly as in our previous works [6,7] (Suppl. Fig. 1). The halves of the samples, which were not used in the contrast agent measurements, were fixed in formalin for two weeks, decalcified in ethylenediaminetetraacetic acid, dehydrated in graded alcohols, and embedded in paraffin. Proteoglycan content was determined with digital densitometry of Safranin-O-stained sections [8]. Collagen content was determined by measuring the absorbance of the amide I region ($1/\lambda$ = 1595 – 1720 cm^{-1}) with Fourier-transform infrared spectroscopy [9]. Polarized light microscopy was used to determine depthwise collagen fiber network orientation and parallelism index (*i.e.*, anisotropy) [10]. For Fourier-transform infrared spectroscopy, polarized light microscopy, and digital densitometry, three sections of each sample were imaged and depthwise profiles were calculated by averaging pixels at the same depth.

For water content analysis, the remaining set of quartered samples were cut into 200 µm sections parallel to the cartilage surface. The depthwise water content was determined by weighing the sections three times to get the mean wet weight and then sections were frozen over-night at -80 °C. Next morning the sections were freeze-dried with a lyophilizator (Alpha 1-2, Martin Christ, Osterode, Germany) and after 24 hours, samples were weighed once more to get the dry weight. Then, the relative water content of each section was calculated by deducting the dry weight from the mean wet weight and comparing it to the mean wet weight. These sections of each sample were then turned into a depthwise profile of 10% segments by linearly interpolating.

Visual and Compositional Differences in Healthy vs. Early Osteoarthritis Cartilage

The differences between healthy (Mankin grade: 0–1.5) and early-stage osteoarthritis (OA) samples (Mankin grade: 1.5–4) are presented in Manuscript Table 2. However, an example of the visual differences between healthy (Mankin grade = 0.3) and early-stage OA (Mankin grade = 3.8) articular cartilage is shown in Supplementary Figure 1. Clear visual differences between these specific samples are observed in proteoglycan content and collagen angle, but not in collagen content. Nevertheless, overall significant differences (between all the healthy and all the early-stage samples) were found in proteoglycan and collagen content, but not in collagen angle or the parallelism index (Manuscript Table 2).

The X-ray attenuation in these same samples was also compared with and without contrast agents in Supplementary Figure 2. Without contrast agents (at the 0-hour timepoint), no observable differences were detected between healthy and early-stage OA cartilage (Supplementary Figures 2A and 2B, and blue lines in Supplementary Figure 2E). In contrast, with contrast agents (at the 96-hour timepoint), clear differences in

attenuation were observed between the samples (Supplementary Figures 2C and 2D, and red lines in Supplementary Figure 2E). Furthermore, this difference became even more pronounced when using material decomposition to separate the contrast agents, providing greater sensitivity in detecting early compositional changes in cartilage (Manuscript Figure 3D and Manuscript Table 2).

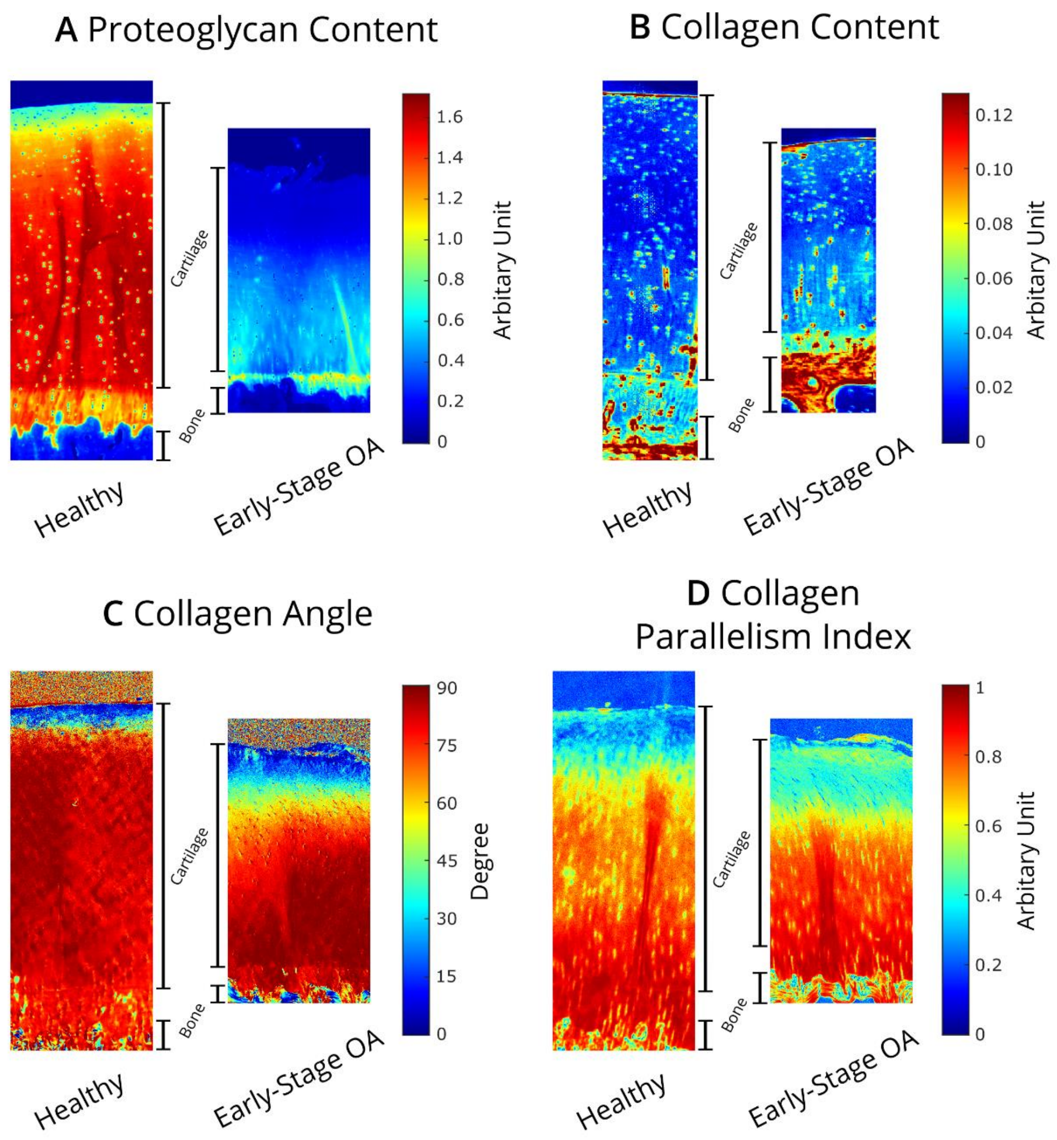

Supplementary Figure 1: Example figure of the reference methods for two samples representing healthy and early-stage osteoarthritis (OA) samples (Mankin grades of the samples: 0.3 and 3.8, respectively). Subfigure (**A**) presents the optical density images showing the proteoglycan content of the samples and subfigure (**B**) shows the Fourier-transform infrared spectroscopy images representing collagen content, where a higher value means higher content. Subfigures (**C**) and (**D**) show polarized light microscopy images for collagen angle (**C**) and parallelism index (**D**), where the 0 degree angle signifies that the collagen fibers are perpendicular to cartilage surface and a higher parallelism index means higher anisotropy between collagen fibers. The differing heights of the subfigures reflect variations in sample thickness, while maintaining a consistent scale.

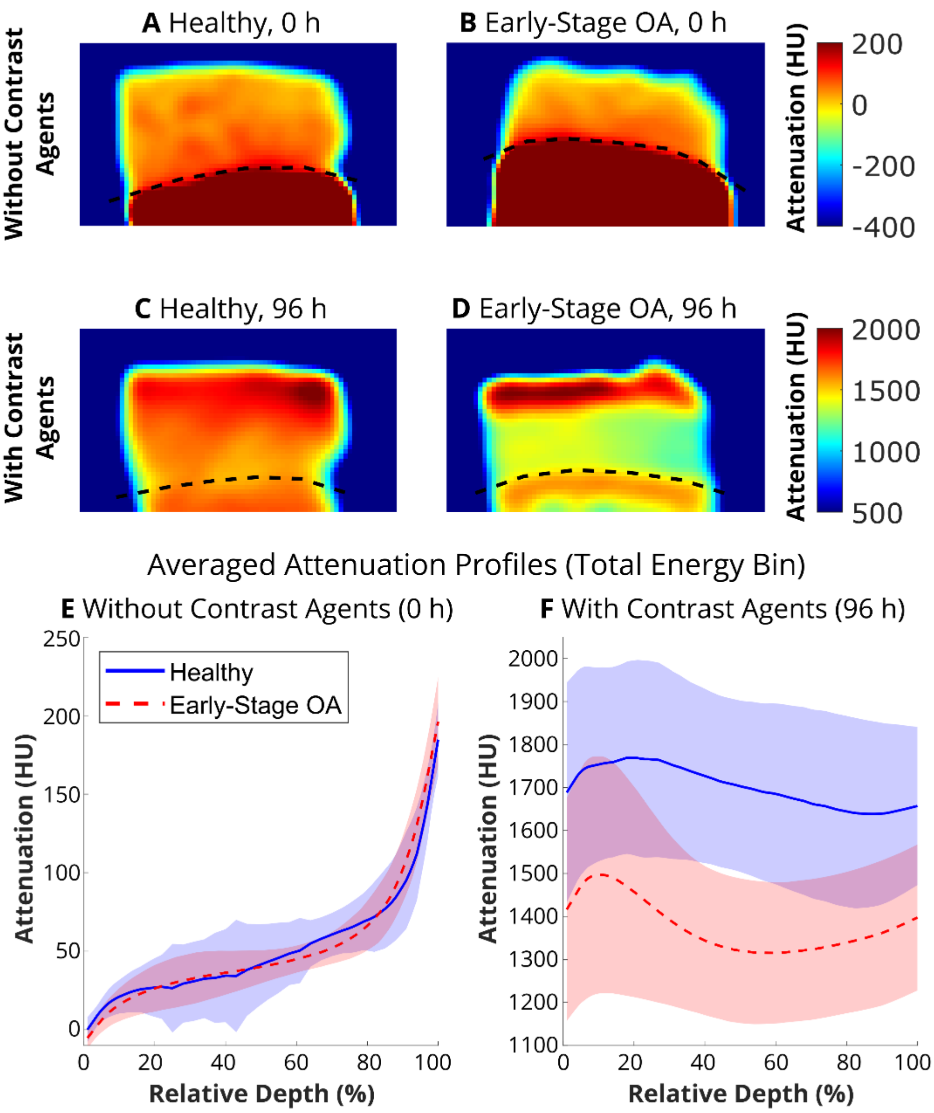

Supplementary Figure 2: X-ray attenuation of two individual samples (one healthy, one early-stage OA) imaged at two different timepoints in the total energy bin of the PCD-CT data. In subfigures **A** and **B** there are PCD-CT scans of healthy and early-stage OA samples without contrast agent at 0-hour timepoint, respectively. In subfigures **C** and **D** there are PCD-CT scans of healthy and early-stage OA samples with contrast agent at 96-hour timepoint, respectively. The averaged attenuation profiles the healthy and early-stage OA sample groups at 0- and 96-hour timepoints are presented in the subfigures **E** and **F**, respectively (solid and dashed lines are the averages and shaded area signify standard deviation). At 0-hour timepoint there are no clear visual differences between the healthy and early-stage OA samples (subfigure **A** and **B**) and the attenuation profiles do not have significant differences (subfigure **E**). However, at 96-hour timepoint, there are visible differences in the attenuation of healthy and early-stage OA articular cartilage (subfigure **C** and **D**) and there are clear differences between the attenuation profiles (subfigure **F**). The attenuation data is acquired from total energy bin of PCD-CT data and is in Hounsfield units (HUs). The dashed black line in subfigures **A-D** signify the cartilage-bone interface and the blue background is air. The articular cartilage thicknesses in subfigures **E** and **F** are linearly interpolated to ensure consistent thickness across all samples, where the cartilage surface is at 0% and the cartilage-bone interface at 100%.

Supplementary Table 1: Properties of the samples in Part A between the sample locations (i.e., distal intertrochlear groove and medial femoral condyle). The lower and upper 95% confidence interval values are given in the brackets in the second row. Statistically significant difference ($p < 0.05$) between the locations (columns with gray background) are indicated with bold typeface. AU = arbitrary unit. P_{96h} = contrast agent partition at 96-hour timepoint.

**Parameter**	**All Samples (*n* = 30)**	**Distal Intertrochlear Groove (*n* = 15)**	**Medial Femoral Condyle (*n* = 15)**
**Mankin Scoring (Grade)**	1.91 ± 1.02<br>[1.53, 2.29]	**2.38 ± 0.74<br>[2.04, 2.71]**	**1.45 ± 1.06<br>[1.11, 1.79]**
**Thickness (mm)**	1.44 ± 0.43<br>[1.28, 1.61]	**1.13 ± 0.28<br>[1.02, 1.24]**	**1.75 ± 0.30<br>[1.64, 1.86]**
**Proteoglycan Content (AU)**	0.87 ± 0.37<br>[0.73, 1.01]	**0.54 ± 0.19<br>[0.46, 0.63]**	**1.19 ± 0.17<br>[1.14, 1.25]**
**Collagen Content (AU)**	25.95 ± 3.92<br>[24.48, 27.41]	26.43 ± 3.78<br>[24.71, 28.15]	25.47 ± 4.13<br>[24.14, 26.79]
**Collagen Angle (°)**	73.61 ± 7.64<br>[70.71, 76.52]	**68.97 ± 7.50<br>[65.56, 72.38]**	**78.59 ± 3.71<br>[77.39, 79.79]**
**Water Content (%)**	71.59 ± 7.98<br>[68.61, 74.57]	**68.70 ± 7.32<br>[65.37, 72.03]**	**74.48 ± 7.76<br>[71.98, 76.98]**
**Equilibrium Modulus, E_{Eq} (MPa)**	0.93 ± 0.68<br>[0.68, 1.19]	**0.45 ± 0.24<br>[0.35, 0.56]**	**1.41 ± 0.65<br>[1.20, 1.62]**
**Dynamic Modulus, E_{Dyn} (MPa)**	4.89 ± 2.21<br>[4.07, 5.72]	**4.03 ± 1.37<br>[3.41, 4.65]**	**5.76 ± 2.58<br>[4.93, 6.59]**
**Phase Shift, θ (°)**	13.17 ± 5.44<br>[11.14, 15.20]	**17.99 ± 2.85<br>[16.70, 19.29]**	**8.35 ± 1.82<br>[7.76, 8.93]**
**Relaxation Time Constant, α (h)**	7.64 ± 6.39<br>[5.21, 10.08]	**2.85 ± 1.29<br>[2.24, 3.46]**	**12.12 ± 5.98<br>[10.19, 14.04]**
**Relaxation Time Constant, β (h)**	0.45 ± 0.10<br>[0.42, 0.49]	**0.41 ± 0.08<br>[0.37, 0.45]**	**0.49 ± 0.10<br>[0.46, 0.52]**
**Iodixanol, P_{96h} (%)**	54.96 ± 5.74<br>[52.82, 57.11]	**57.39 ± 4.13<br>[55.51, 59.27]**	**52.54 ± 6.23<br>[49.71, 55.37]**
**Ta_2O_5-cNP, P_{96h} (%)**	214.48 ± 90.08<br>[180.84, 248.14]	**172.25 ± 54.30<br>[147.56, 196.94]**	**256.71 ± 100.18<br>[211.15, 302.27]**
**Combined Partition, P_{96h} (%)**	399.42 ± 183.26<br>[330.99, 467.85]	**298.30 ± 84.42<br>[259.91, 336.69]**	**500.54 ± 201.32<br>[408.98, 592.09]**

References

[1] Jakubek J, Vavrik D, Pospisil S, Uher J. Quality of X-ray transmission radiography based on single photon counting pixel device. Nucl Instrum Methods Phys Res A 2005;546:113–7. https://doi.org/10.1016/j.nima.2005.03.045.

[2] Juntunen MAKK, Inkinen SI, Ketola JH, Kotiaho A, Kauppinen M, Winkler A, et al. Framework for Photon Counting Quantitative Material Decomposition. IEEE Trans Med Imaging 2020;39:35–47. https://doi.org/10.1109/TMI.2019.2914370.

[3] van Aarle W, Palenstijn WJ, De Beenhouwer J, Altantzis T, Bals S, Batenburg KJ, et al. The ASTRA Toolbox: A platform for advanced algorithm development in electron tomography. Ultramicroscopy 2015;157:35–47. https://doi.org/10.1016/j.ultramic.2015.05.002.

[4] van Aarle W, Palenstijn WJ, Cant J, Janssens E, Bleichrodt F, Dabravolski A, et al. Fast and flexible X-ray tomography using the ASTRA toolbox. Opt Express 2016;24:25129. https://doi.org/10.1364/OE.24.025129.

[5] Paakkari P, Inkinen SI, Honkanen MKM, Prakash M, Shaikh R, Nieminen MT, et al. Quantitative dual contrast photon-counting computed tomography for assessment of articular cartilage health. Sci Rep 2021;11:5556. https://doi.org/10.1038/s41598-021-84800-x.

[6] Fugazzola M, Nissinen MT, Jäntti J, Tuppurainen J, Plomp S, Te Moller N, et al. Composition, architecture and biomechanical properties of articular cartilage in differently loaded areas of the equine stifle. Equine Vet J 2023:1–13. https://doi.org/10.1111/evj.13960.

[7] Jäntti J, Joenathan A, Fugazzola M, Tuppurainen J, Honkanen JTJ, Töyräs J, et al. Cationic tantalum oxide nanoparticle contrast agent for micro computed tomography reveals articular cartilage proteoglycan distribution and collagen architecture alterations. Osteoarthritis Cartilage 2024;32:299–309. https://doi.org/10.1016/j.joca.2023.11.020.

[8] Kiviranta I, Jurvelin J, Tammi M, Saamanen AM, Helminen HJ. Microspectrophotometric quantitation of glycosaminoglycans in articular cartilage sections stained with Safranin O. Histochemistry 1985;82:249–55.

[9] Saarakkala S, Julkunen P, Kiviranta P, Mäkitalo J, Jurvelin JS, Korhonen RK. Depth-wise progression of osteoarthritis in human articular cartilage: investigation of composition, structure and biomechanics. Osteoarthritis Cartilage 2010;18:73–81. https://doi.org/10.1016/j.joca.2009.08.003.

[10] Rieppo J, Hallikainen J, Jurvelin JS, Kiviranta I, Helminen HJ, Hyttinen MM. Practical considerations in the use of polarized light microscopy in the analysis of the collagen network in articular cartilage. Microsc Res Tech 2008;71:279–87. https://doi.org/10.1002/jemt.20551.
